# Design, recruitment, and baseline characteristics of the LENS trial

**DOI:** 10.1111/dme.15310

**Published:** 2024-02-22

**Authors:** The LENS Collaborative Group, David Preiss, Jennifer Logue, Emily Sammons, Mohammed Zayed, Jon Emberson, Rachel Wade, Karl Wallendszus, Will Stevens, Simon Harding, Graham Leese, Gemma Currie, Jane Armitage

**Affiliations:** 1 Clinical Trial Service Unit and Epidemiological Studies Unit, Nuffield Department of Population Health, University of Oxford, Oxford; 2 Medical Research Council Population Health Research Unit at the University of Oxford; 3 Faculty of Health and Medicine, University of Lancaster, Lancaster; 4 Department of Eye and Vision Science, University of Liverpool and St. Paul’s Eye Unit, Liverpool University Hospitals NHS Foundation Trust, Liverpool; 5 Molecular and Clinical Medicine, University of Dundee, Dundee; 6 School of Cardiovascular & Metabolic Health, University of Glasgow, Glasgow

**Keywords:** fenofibrate, diabetic retinopathy, randomised trial

## Abstract

**Background:**

Findings from cardiovascular outcome trials suggest that treatment with fenofibrate may reduce the progression of diabetic retinopathy. However, no dedicated large-scale randomised trials have yet investigated this hypothesis.

**Methods:**

LENS is a streamlined randomised double-masked placebo-controlled trial, based in Scotland, assessing whether treatment with fenofibrate (145mg tablet daily or, in the context of impaired renal function, on alternate days) in people with early retinopathy reduces progression to referable diabetic retinopathy (defined in NHS Scotland’s Diabetic Eye Screening grading scheme as referable background or proliferative retinopathy, or referable maculopathy in either eye) or treatment with retinal laser, intra-vitreal injections or vitrectomy. Adults with diabetes mellitus and non-referable retinopathy (mild background retinopathy in both eyes or observable background retinopathy in one/both eyes at the most recent NHS retinal screening assessment; or observable maculopathy in one/both eyes in the previous 3 years) were eligible. Potential participants were identified from routinely collected healthcare data, and followed up using regular contact from the research team and linkage to national electronic morbidity, mortality, biochemistry and retinal screening records. Study treatment was mailed to participants.

**Results:**

Between 18 September 2018 and 27 July 2021, 1151 participants were randomised. Their mean age was 61 (SD 12) years, 312 (27%) were female, and 305 (26%) had type 1 diabetes. 96% had bilateral mild background retinopathy and 10% had observable maculopathy.

**Conclusions:**

LENS will provide a robust evaluation of the efficacy of treating people at risk of progression of diabetic retinopathy with fenofibrate. Results are anticipated in mid-2024.

**Trial registrations:**

NCT03439345; ISRCTN15073006; EuDRACT 2016-002656-24

Diabetic retinopathy (DR) is a common progressive microvascular complication of diabetes mellitus and a leading cause of visual loss^1^, one of the most feared complications of diabetes.^2^ Non-proliferative disease is characterised by changes in the retina including microaneurysms, dot and blot haemorrhages, exudates, cotton wool spots, venous changes and intraretinal microvascular abnormalities. Progression to proliferative disease may cause complications including vitreous haemorrhage, retinal detachment and neovascular glaucoma, resulting in visual impairment. Diabetic maculopathy is characterised by microaneurysms, exudates, and/or haemorrhages within the macula, which may progress to macular oedema and also threaten vision.

Key risk factors for the progression of DR include longer duration of diabetes, higher HbA1c and hypertension.^3^ There are few effective options to slow the progression of DR. Lowering blood pressure modestly reduces the progression of DR.^4^ Landmark trials conducted before 2000 also showed that moderately good glycaemic control substantially reduced DR complications compared with poor glycaemic control.^5,6^ However, more recent trials comparing intensive with less intensive glucose-control yielded only modest benefit.^7^ Treatments for sight-threatening disease, such as retinal laser and intra-vitreal injections, are expensive, associated with various risks and not effective in every patient. Therapies that are beneficial earlier in the disease and are more cost-effective are therefore needed to reduce the chances of people with diabetes developing progressive DR.

Fenofibrate is a peroxisome proliferator-activated receptor alpha agonist that reduces circulating triglycerides and LDL-cholesterol, and increases HDL-cholesterol.^8^ Two randomised placebo-controlled cardiovascular outcome trials of fenofibrate therapy, conducted in participants with type 2 diabetes, have reported on DR outcomes. In the Fenofibrate Intervention and Event Lowering in Diabetes (FIELD) study, adjudicated reports of retinal laser therapy, a tertiary outcome, were proportionally reduced by 31% (164 first events in 4895 (3.4%) participants on fenofibrate vs. 238 first events in 4900 (4.9%) participants on placebo^9,10^). The Action to Control Cardiovascular Risk in Diabetes (ACCORD) Lipid study was conducted in 5518 participants on background open-label simvastatin.^11^ 1593 ACCORD Lipid participants joined the ACCORD Eye sub-study, which included two standardised eye examinations and fundal photography at baseline and 4 years.^12^ The composite outcome of laser treatment, vitrectomy or 3-step progression on the Early Treatment Diabetic Retinopathy Study (ETDRS) scale was proportionally reduced by 40% (52 first events in 806 (6.5%) participants on fenofibrate vs. 80 first events in 787 (10.2%) participants on placebo).

Although promising, these results should be considered hypothesis-generating because they are derived from analyses of non-primary trial outcomes in null cardiovascular trials and do not take account of multiple statistical testing. There is therefore a pressing need for trials designed with the primary intention of investigating the effect of fenofibrate on the progression of DR. The Lowering Events in Non-proliferative retinopathy in Scotland (LENS) trial is designed to achieve this objective, making use of existing healthcare data to recruit and follow participants. In this manuscript, we describe the design, recruitment and baseline characteristics of the LENS trial.

## Materials and Methods

### Trial organisation

LENS was designed by investigators based at the trial’s Central Coordinating Office (CCO) at the Clinical Trial Service Unit and Epidemiological Studies Unit, University of Oxford. It is run by the Trial Management Group at the CCO who work closely with the Regional Coordinating Centre at NHS Greater Glasgow and Clyde to guide activities at sites within all 11 mainland NHS Scotland Health Boards. The trial has oversight from a majority independent Trial Steering Committee (TSC), constituted from clinical experts and patient representatives. An independent Data Monitoring Committee (DMC) is responsible for reviewing unmasked data every 6 months to assess participant safety and trial progress, and for making recommendations to the TSC. The University of Oxford is the trial sponsor.

### Aims

The LENS trial set out to randomise participants with non-referable DR to fenofibrate or placebo to assess its effect on time to the primary composite outcome of progression to referable DR or treatment for DR. Referable DR is defined as referable background (sometimes called severe non-proliferative) DR or proliferative DR or referable maculopathy in either eye (Table 1). Referable DR in the trial is typically identified during NHS retinal screening but may also be identified from adverse event reports. Treatment for DR includes retinal laser therapy, intra-vitreal injection and vitrectomy. Analyses of change in visual acuity, visual function, quality of life and health economics are also planned along with other pre-specified eye outcomes. The primary, secondary and tertiary outcomes are listed in Table 2.

**Figure 1:**
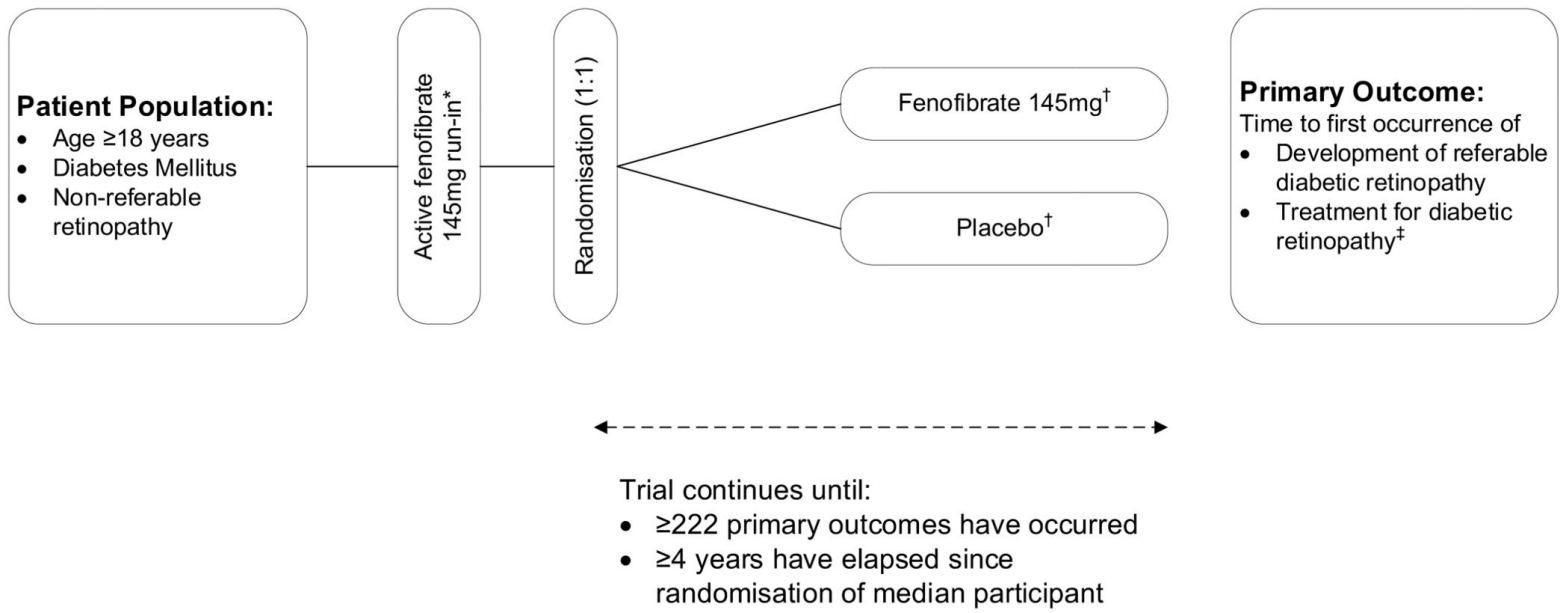
Design of the LENS trial *Footnote*: * One tablet daily if eGFR ≥60 mL/min/1.73m^2^, one tablet on alternate days if 40-59 mL/min/1.73m^2^; ^†^ one tablet daily if eGFR ≥60 mL/min/1.73m^2^, one tablet on alternate days if 30-59 mL/min/1.73m^2^; ^‡^ defined as retinal laser, intra-vitreal injection or vitrectomy for diabetic eye disease

The design of LENS is streamlined and heavily embedded within routine clinical care. Data collection consists of participant-reported information being recorded directly into a bespoke web-based system during study assessments alongside regular linkage to NHS Scotland healthcare datasets. The protocol is included in the supplementary materials and the design is summarised in Figure 1.

### Retinal screening in Scotland

Retinal screening in Scotland is routinely offered to people with diabetes. Single 45 degree retinal photographs of each eye showing the macula and optic disc are taken through an undilated pupil. Mydriatic eye drops are used if undilated images are inadequate. When adequate images are still not obtained, slit lamp examination is arranged. Retinal images are graded according to the NHS Scotland Diabetic Eye Screening (DES) scheme (Table 1) and undergo three levels of grading – Level 1 (typically conducted using image analysis software) to identify images with retinal disease, Level 2 (by junior graders) to identify images with potentially sight-threatening disease, and Level 3 (by senior graders) to make the final decision regarding which patients require specialist referral or further investigation. Graders participate in an annual external quality assurance programme.

### Healthcare datasets in Scotland

The Community Health Index (CHI) number is a unique 10-character numeric identifier allocated to each NHS Scotland patient. This allows linkage to healthcare datasets including:

- *Diabetic Eye Screening (DES)*: retinal images and results from retinal screening records
- *Scottish Care Information – Diabetes (SCI Diabetes)*: integrated electronic patient record used in general practice and hospitals to support treatment of diabetes, including biochemistry and retinal screening data
- *National Records of Scotland (NRS) death registrations*
- *Prescribing Information System (PIS)*: data regarding medicines prescribed in the community
- *General Acute Inpatient and Day Case – Scottish Morbidity Record (SMR01)*: data regarding hospitalisations (for health economic analyses)
- *Outpatient Appointments and Attendances – Scottish Morbidity Record (SMR00)*: data for outpatient hospital appointments (for health economic analyses)

### Eligibility

Consenting adults with diabetes mellitus (other than gestational diabetes) and non-referable DR were potentially eligible to join the trial. Non-referable DR is defined as: (a) mild background DR (R1) in both eyes or observable background DR (R2) in one/both eyes at the most recent NHS retinal screening assessment, or (b) observable maculopathy (M1) in one/both eyes at an NHS retinal screening assessment in the last 3 years (Table 1; it should be noted that participants were invited based on non-referable DR at their most recent retinal screening and not earlier retinal screening results). We excluded patients with no DR or only unilateral mild background DR as they are at low risk of progression to referable DR^13^ (having, respectively, approximately 15-fold and 4-fold lower risks than the trial’s target population based on DES data (available in the protocol)). Hepatobiliary diseases, renal replacement therapy, previous organ transplant, significant muscle disease, pregnancy, breastfeeding and the use of certain medications were the other main exclusion criteria. Selected biochemical tests were also conducted at screening and randomisation assessments to assess eligibility. Estimated glomerular function rate (eGFR) was required to be ≥40 mL/min/1.73m^2^ at screening and ≥30 mL/min/1.73m^2^ at the randomisation assessment to allow for the expected increase in serum creatinine on fenofibrate during the active run-in.^14,15^ There were no limits on lipids, HbA1c or blood pressure to enter the trial. Table 3 provides the full eligibility criteria.

### Invitation, pre-screening, screening, run-in and randomisation

- *Invitation*: with appropriate approvals, SCI Diabetes staff performed searches to identify adults in mainland Scotland with diabetes who had non-referable DR at their most recent retinal screening and eGFR ≥40 mL/min/1.73m^2^ (but not other eligibility criteria). These lists were provided securely to NHS Scotland Health Boards for approval, following which they were sent to the Health Informatics Centre (HIC), University of Dundee. HIC mailed invitation letters (including a Participant Information Leaflet and reply slip) to potentially eligible individuals.
- *Pre-screening*: Study site staff used available medical records to perform pre-screening for interested respondents based on retinal screening results, renal function, and any other obvious exclusion criteria.
- *Screening assessment*: At this in-person visit, information regarding eligibility, other relevant clinical information and contraindicated medications was recorded, and informed consent was obtained. Participants completed Quality of Life (EQ-5D-5L) and Visual Function (VFQ-25) questionnaires on paper. Samples were sent to local NHS biochemistry laboratories for analyses of renal function, liver function tests, creatine kinase, HbA1c, random lipid profile and urine albumin: creatinine ratio.
- *Active run-in*: Site investigators (or delegated study doctors) electronically approved eligible participants’ entry into an active run-in phase. Participants were then mailed a 10-week supply of open-label nanoparticle fenofibrate 145mg tablets. Those with screening eGFR ≥60 mL/min/1.73m^2^ were instructed to take one tablet daily and those with eGFR 40-59 mL/min/1.73m^2^ to take one tablet on alternate days.
- *Randomisation assessment*: Participants were seen in person approximately 8 weeks later. Compliance with the run-in treatment, concomitant medications and adverse events during run-in were documented. Participants who reported poor compliance (i.e. taking fewer than five tablets per week for those assigned daily treatment and taking less than five tablets every two weeks for those assigned alternate day treatment) were excluded. Height, weight, pulse rate and blood pressure were recorded. For eligible participants who were willing to proceed, a blood sample was taken to measure renal function, liver function tests and creatine kinase. When sites were temporarily closed due to the COVID-19 pandemic, site staff were permitted to perform randomisation assessments by telephone; however, blood results were still required to fully assess eligibility and samples were collected at the soonest opportunity thereafter.
- *Randomisation*: A web-based minimisation algorithm was used to randomise participants in a 1:1 ratio between fenofibrate and placebo. Minimisation criteria (listed in the protocol) were selected to balance prognostically important variables.^16^ The randomisation algorithm also contained a stochastic element, with simple randomisation used 10% of the time.
- *Follow up*: Trial-specific questionnaires were administered by telephone approximately every six months to obtain information about adverse events leading to cessation of study treatment, all serious adverse events, eye outcome events and the use of contraindicated medications. There was an option for follow up via medical record review if a participant could not be contacted or no longer wished to be contacted. Paper EQ-5D-5L and VFQ-25 questionnaires were mailed to participants for completion 2 years after randomisation and at the end of the trial. Participants continuing study treatment were regularly mailed 26 week supplies of nanoparticle fenofibrate 145mg or placebo. The appropriate dose of fenofibrate depended on renal function. Those with a randomisation assessment eGFR ≥60 mL/min/1.73m^2^ were commenced on one tablet daily and those with eGFR 30-59 mL/min/1.73m^2^ on one tablet on alternate days. Because no face-to-face follow-up study visits were conducted after randomisation during the trial, eGFR results were monitored remotely using blood results from routine care (noting that >90% of patients with diabetes in Scotland have their renal function checked every 15 months^17^). A study doctor at the Regional Coordinating Centre performed monthly automated searches of SCI Diabetes to identify participants with post-randomisation eGFR falling to 30-59 mL/min/1.73m^2^ (study drug was then reduced to one tablet on alternate days) or to <30 mL/min/1.73m^2^ (study treatment was then stopped but could be restarted if renal function improved). Linkages to healthcare datasets were performed regularly including DES and SCI Diabetes (to identify pre-specified DR outcomes), and to SMR01, SMR00, PIS and the NRS death registry.
- *Adjudication*: Adverse event reports of eye procedures, vitreous haemorrhages and macular oedema were adjudicated by experienced doctors at the CCO, masked to treatment allocation.

### Statistical considerations

DES data suggested that progression from non-referable to referable DR in the target population would occur in approximately 29% of patients over 4 years. We calculated that a sample size of 1060 participants would provide 85% power (at 2-sided p<0.05) to detect a 33% reduction in the primary outcome, based on 222 first events occurring over an expected average follow-up period of 4 years (allowing for 15% drop-out e.g. no longer attending retinal screening). The trial was therefore designed to continue until two conditions were met: (i) the occurrence of at least 222 primary outcome events; and (ii) at least 4 years to have elapsed from randomisation of the median participant. No formal interim analyses were planned for early stopping due to efficacy. The statistical analysis plan is included in the supplementary materials.

## Results

### Recruitment

**Figure 2:**
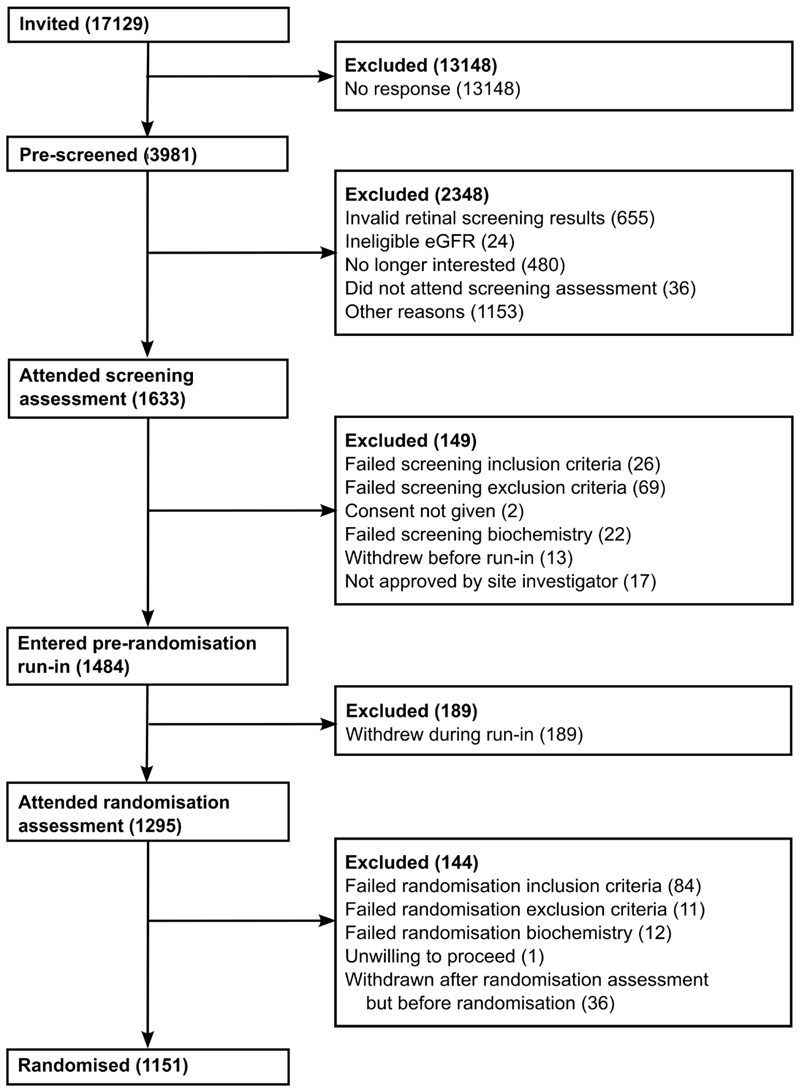
Trial profile – flow of participants through LENS trial recruitment

17,129 individuals were invited to participate across mainland Scotland. Of them, 3981 (23.2%) responded positively and were pre-screened with 2348 excluded before screening (655 due to ineligible retinal screening results, 24 due to low eGFR, 480 who were no longer interested when contacted, 1153 who were ineligible for other reasons and 36 who were eligible but did not attend the screening visit). 1633 attended a trial screening assessment. Of them, 1484 entered the active run-in phase and 149 did not (117 failed the eligibility criteria, 2 declined to give consent, 13 withdrew during screening and 17 were not approved to enter the run-in phase by the site investigator). A further 189 participants withdrew during the run-in period. Of the 1295 participants who attended a randomisation assessment, 144 were ineligible (107 failed the eligibility criteria and 37 were withdrawn after the randomisation assessment but before randomisation, typically based on new retinal screening results). From 18 September 2018 to 27 July 2021, 1151 participants were randomised (Figure 2).

### Characteristics of 1151 randomised LENS participants

Mean (standard deviation) age was 60.7 (12.3) years and 839 (73%) were men. There were 305 (26%) participants with type 1 diabetes. Mean total cholesterol was 4.0 (1.0) mmol/L and 854 (74%) were on statin therapy. Mean HbA1c was 66 (16) mmol/mol (8.2 (1.5) %) and participants had a long duration of diabetes (18 (10) years). The substantial majority (96%) had bilateral mild background DR (R1), and 10% had observable maculopathy (M1) in at least one eye at their most recent retinal screening visit. Serum creatinine rose from the screening assessment to the randomisation assessment due to the known effect of fenofibrate. As a consequence, the proportion of those with eGFR <60 mL/min/1.73m^2^ increased from 9% at the screening assessment to 23% at the randomisation assessment (Table 4).

To assess the representativeness of LENS participants, SCI Diabetes data were used to compare them to the potentially eligible population in Scotland. This was achieved by repeating the SCI Diabetes recruitment search (based on age ≥18 years, non-referable DR and eGFR (as used to determine LENS eligibility)) in August 2023. Participants recruited into LENS were broadly representative of the population of patients in Scotland, identified as potentially eligible for the trial, with respect to important risk factors for DR progression including age, type of diabetes, HbA1c, DR grading and eGFR (Table 5). The proportions of women and individuals of non-White ethnicity were lower in LENS than in the potentially eligible population. Based on the Scottish Index of Multiple Deprivation (SIMD), a measure of social deprivation based on home address post code^18^, trial participants were modestly less socially deprived than the target population.

### COVID-19 and retinal screening

The COVID-19 pandemic led to temporary cessation of the national retinal screening programme in April 2020. When NHS Scotland restarted retinal screening, it was necessary to apply new procedures for cleaning equipment and spacing, thereby slowing the throughput for retinal screening for a period of time. However, activity has subsequently increased and retinal screening activity in LENS participants has returned to full capacity.

### Follow up

The median participant was randomised on 16 July 2019. Given that masked assessments showed that the primary outcome event rate was at least as high as assumed in the sample size calculation (despite the COVID-19 pandemic), final follow up assessments commenced on 17 July 2023. Final follow up assessments and final data linkages occurred in late 2023.

## Discussion

The LENS trial is designed to assess the efficacy of treatment with fenofibrate for slowing the progression of DR in 1151 people with diabetes and non-referable DR, using a streamlined design. The trial is heavily embedded within routine NHS care and uses retinal screening results (to both invite potentially eligible patients and identify pre-specified DR outcomes) and regular telephone calls to participants to record clinical events of interest.

Large placebo-controlled cardiovascular outcome trials of fenofibrate have suggested that treatment for 4-5 years may substantially reduce the progression of DR.^9,10,12^ We designed LENS to investigate this hypothesis, using nanoparticle fenofibrate, a formulation not influenced by food intake.^19^ Other trials, namely FAME-1-EYE (recruiting participants with type 1 diabetes and non-proliferative DR; NCT01320345) and Fenofibrate for Prevention of DR Worsening (recruiting participants with non-proliferative DR; NCT04661358) are also investigating the effect of fenofibrate on DR for which there is accumulating evidence of a direct effect on the retina^20^. Unlike LENS, which links to NHS Scotland’s retinal screening programme, these two trials use ETDRS-graded retinal images collected at set time points for their main outcomes. LENS participants were asked for consent for their retinal screening images to be securely stored in an imaging biobank at the CCO, University of Oxford, for subsequent research. Approximately 9000 images from 4000 retinal screening episodes had been received as of July 2023. This will facilitate grading using other systems and, therefore, pooling of data with other fenofibrate trials in the future.

Comparison of LENS trial participants with all potentially eligible participants (based on recent retinal screening and eGFR ≥40 mL/min/1.73m^2^) in mainland Scotland shows that they are highly representative with respect to important risk factors for progressive DR. For example, over a quarter of participants have type 1 diabetes, in keeping with the high risk of incident and progressive DR in such individuals. However, 27% of LENS participants are women, compared with 39% of potentially eligible participants. There are two likely contributors. First, the LENS Participant Information Leaflet stressed the lack of safety information in pregnancy and breastfeeding, and explained that pre-menopausal women must use reliable contraception. This is likely to have dissuaded some women from joining the trial. Second, information regarding other randomised trials conducted in cardio-metabolic disease shows that women appear less likely to volunteer.^21^ Trial participants are also modestly less socially deprived, based on SIMD score, a pattern observed in other trials.^21^ The substantial majority (98%) of LENS participants are white, similar to other large diabetes trials in the UK^22^, and more than the estimated proportion (approximately 91% of potentially eligible individuals with race recorded) in Scotland.^17^

Retinal screening programmes exist in various healthcare settings. These programmes serve to identify patients with sight-threatening disease so that they can receive specialist follow up and, if required, treatment. LENS suggests that this infrastructure also provides a useful setting to identify those at higher risk of progression at an earlier stage (if efficacious treatment options can be identified) and to conduct randomised trials. An additional advantage of conducting LENS in a country with a single provider of retinal screening is that it offers the opportunity for prolonged post-trial follow up, and trial participants provided consent for this. The design proved to be generally robust to the impact of the COVID-19 pandemic – we were able to conduct follow up assessments by telephone and to send study treatment to participants by mail.

In summary, LENS will provide a robust evaluation of the efficacy of fenofibrate treatment in a representative population of people with non-referable DR. Results from the trial are expected in mid-2024.

## Supplementary Material

Supplement

## Figures and Tables

**Table 1:** Scottish Diabetic Eye Screening grading scheme for diabetic retinopathy

Grading	Description	Findings	Outcome
**RETINOPATHY (excluding the macula)**			
R0	No DR anywhere	-	Rescreen in 12-24 months
R1	Mild background diabetic retinopathy	The presence of at least one of any of the following features anywhere: dot haemorrhages / microaneurysms hard exudates cotton wool spots blot haemorrhages* superficial/ flame shaped haemorrhages	Rescreen in 12 months
R2	Observable background diabetic retinopathy	Four or more blot haemorrhages* in one hemi-field only (Inferior and superior hemi-fields delineated by a line passing through the centre of the fovea and optic disc)	Rescreen in 6 months
R3	Referable background diabetic retinopathy	Any of the following features: four or more blot haemorrhages* in both inferior and superior hemi-fields Venous beading Intraretinal Microvascular Abnormalities (IRMA)	Specialist referral (routine)
R4	Proliferative diabetic retinopathy	Any of the following features: Active new vessels Vitreous haemorrhage	Specialist referral (urgent)
R4i^†^	Treated proliferative diabetic retinopathy	Any of the following features: Inactive new vessels with evidence of laser treatment	Rescreen in 12 months
R6	Not adequately visualised	Retina not sufficiently visible for assessment	Technical failure
**MACULOPATHY**			
M0	No maculopathy	No features ≤2 disc diameters from the centre of the fovea sufficient to qualify for M1 or M2	Rescreen in 12-24 months
M1	Observable maculopathy	Lesions as specified below within a radius of >1 but ≤2 disc diameters from the centre of the fovea: Any hard exudates	Rescreen in 12 months
M2^‡^	Referable maculopathy	Lesions as specified below within a radius of ≤1 disc diameter of the centre of the fovea: Any blot haemorrhages* Any hard exudates	OCT surveillance scan or Rescreen in 12 months

* blot haemorrhage has the same or greater diameter as a retinal vein crossing the optic disc

† R4i is not counted towards the primary outcome as it represents inactive disease

‡ At the start of LENS in 2018 and until end-2021, all M2 results led to specialist ophthalmology referral. During 2022, DES started to introduce a phased change to the management pathway of patients with M2 disease. In the new pathway, patients graded M2 with poor visual acuity (Snellen 6/9.5 or worse) are referred for optical coherence tomography (OCT) imaging. If this shows central referable macular oedema, the patient is referred to an ophthalmologist, with medical retina expertise for further assessment and treatment. Patients who do not require OCT, or in whom there is no evidence of central referable macular oedema on OCT, are not automatically referred to a specialist. Given that M2 (or equivalent disease) requires specialist referral/review in many countries, LENS continued to categorise M2 as referable maculopathy after this change.

**Table 2:** Primary and secondary outcomes for the LENS trial

Primary outcome
A composite of the development of referable diabetic retinopathy (defined as referable background retinopathy or proliferative retinopathy or referable maculopathy in either eye) or treatment for diabetic retinopathy (including retinal laser therapy, vitrectomy or intra-vitreal injection of medication)
**Secondary outcomes**
The composite primary outcome in various subgroups: ○ sex (men vs. women) ○ age (<60 years vs. ≥60 years ○ type of diabetes (type 1 diabetes vs. type 2 diabetes and other types) ○ renal function (randomisation eGFR <60 vs. ≥60 mL/min/1.73m^2^) ○ HbA1c <70 mmol/mol (8.6%) vs. ≥70 mmol/mol (8.6%) ○ first primary outcome in the first year vs. subsequent years of follow-up The individual components of the composite primary outcome, namely: ○ the development of referable diabetic retinopathy ○ treatment for diabetic retinopathy: any of retinal laser therapy, vitrectomy or intra- vitreal injection Any progression of diabetic retinopathy The presence of hard exudates or blot haemorrhages within 1 disc diameter of the macula Macular oedema* Change in visual function Change in quality of life Change in visual acuity Health economic assessments
**Tertiary outcomes**
Change in urine albumin: creatinine ratio Major cardiovascular events (composite of myocardial infarction, stroke, coronary and peripheral arterial revascularisation) Non-traumatic lower limb amputations

* based on (i) any finding of macular oedema during OCT imaging as part of the DES retinal screening programme, and (ii) adjudicated adverse event reports of macular oedema

**Table 3:** Inclusion and exclusion criteria for entry into the LENS trial

Inclusion criteria
All of the following criteria must be fulfilled: Subjects capable of giving informed consent Diabetes Mellitus (any type except gestational diabetes) Age ≥18 years Non-referable diabetic retinopathy (defined according to NHS Scotland’s Diabetic Eye Screening grading scheme as mild background retinopathy (R1) in both eyes or observable background retinopathy (R2) in one/both eyes at the most recent NHS retinal screening assessment; or observable maculopathy (M1) in one/both eyes at any NHS retinal screening assessment in the last 3 years) Willing to either complete electronic questionnaires or conduct telephone interviews for collection of questionnaire data once every 6 months
**Exclusion criteria**
None of the following criteria must be fulfilled: Referable diabetic retinopathy (defined by NHS Scotland’s Diabetic Eye Screening grading scheme as referable background retinopathy (R3) or proliferative retinopathy (R4) or referable maculopathy (M2) in either eye) History of gallbladder disease (cholecystitis, symptomatic gallstones, cholecystectomy) History of acute or chronic pancreatitis Alanine aminotransferase (ALT) or aspartate aminotransferase (AST) >2X the upper limit of normal (ULN) at screening visit; ALT or AST >2.5X ULN at randomisation visit Creatine kinase (CK) >3X ULN at screening visit; CK >3X ULN at randomisation visit Estimated glomerular filtration rate (eGFR) <40 mL/min/1.73m^2^ at screening visit; eGFR <30 mL/min/1.73m^2^ at randomisation visit Cirrhosis of any aetiology or any other serious hepatic disease Female who is pregnant, breastfeeding, currently trying to become pregnant, or of child-bearing potential and not practising birth control Ongoing vitamin K antagonist (warfarin, phenindione, acenocoumarol), cyclosporine, colchicine, ketoprofen, daptomycin, fibrate therapy, or treatment with rosuvastatin 40 mg daily Previous myositis, myopathy or rhabdomyolysis of any cause, or diagnosed hereditary muscle disorder Ongoing renal replacement therapy Any previous organ transplant Previous reported intolerance to any fibrate Medical history that might limit the individual’s ability to take trial treatments for the duration of the study (e.g. severe respiratory disease, history of cancer within last 5 years other than non-melanoma skin cancer; or recent history of alcohol or substance misuse) Any other significant disease or disorder which, in the opinion of the Investigator, may either put the participant at risk because of participation in the trial, or may influence the result of the trial, or the participant’s ability to participate in the trial Participation in any other study or trial that excludes co-enrolment or if the intervention being investigated in another trial has the potential to interact with fenofibrate therapy Not adherent to active run-in treatment

**Table 4:** Baseline characteristics of LENS participants

	Overall (N=1151)	
**DEMOGRAPHICS**		
**Age at randomization (years)**	60.7	(12.3)
**Sex**		
Male	839	(73%)
Female	312	(27%)
**Ethnicity**		
White	1125	(98%)
Other	26	(2%)
**PRIOR DISEASE**		
**Type of diabetes mellitus**		
Type 1	305	(26%)
Type 2	844	(73%)
Other	2	(0%)
**Duration of diabetes (years)**	18.0	(10.3)
Retinopathy grading (worse eye)*		
No retinopathy (R0)	9	(1%)
Mild background retinopathy (R1)	1126	(98%)
Observable background retinopathy (R2)	16	(1%)
Maculopathy grading (worse eye)*^†^		
No maculopathy (M0)	1032	(90%)
Observable diabetic maculopathy (M1)	119	(10%)
**CLINICAL MEASUREMENTS**		
**Systolic blood pressure (mmHg)^§^**	136.6	(17.5)
**Diastolic blood pressure (mmHg)^§^**	75.5	(9.4)
**Body mass index (kg/m^2^)^§^**	30.8	(6.2)
**LABORATORY MEASUREMENTS**		
**HbA1c (mmol/mol)^§^**	66	(16)
**HbA1c (%)^§^**	8.2	(1.5)
**Total cholesterol (mmol/L)^§^**	4.0	(1.0)
**HDL cholesterol (mmol/L)^§^**	1.3	(0.4)
**Triglycerides (mmol/L)** ^‡^ **^§^**	1.5	(1.1-2.3)
**Serum Creatinine (umol/L)^¶^**		
Screening assessment	77.9	(18.5)
Randomisation assessment	89.1	(22.3)
**eGFR (mL/min/1.73m^2^)^¶^**		
**Screening assessment**		
<60	98	(9%)
≥60	1053	(91%)
**Randomisation assessment**		
<60	261	(23%)
≥60	890	(77%)
**CONCOMITANT MEDICATIONS**		
**Non-insulin glucose-lowering therapy**	785	(68%)
**Insulin**	508	(44%)
**Statin**	854	(74%)
**Renin angiotensin system inhibitor**	686	(60%)

Results are shown as N (%) or mean (standard deviation) unless otherwise indicated.

* Worse eye retinopathy defined as R2 > R1 > R0; worse eye maculopathy defined as M1 > M0.

† 16 participants had bilateral M0 and either bilateral R0 or R1/R0 at their most recent retinal screening prior to randomisation, and therefore qualified based on M1 from an earlier retinal screening visit within the last 3 years

‡ Median (IQR).

¶ Fenofibrate therapy is known to reversibly increase serum creatinine and reduce eGFR.

§ Missing data: systolic and diastolic blood pressure 70 participants, BMI 35 participants, HbA1c 74 participants, total cholesterol 12 participants, HDL cholesterol 23 participants, triglycerides 17 participants (no missing data for other variables)

**Table 5:** Representativeness of LENS trial participants

	Potentially eligible people*		LENS participants	
**N**	31118		1151	
**Age at randomization (years)**	60.5	(15.3)	60.7	(12.3)
**Sex**				
Male	18958	(61%)	839	(73%)
Female	12160	(39%)	312	(27%)
**Ethnicity^†^**				
White	24001	(91%)	1125	(98%)
Other	2475	(9%)	26	(2%)
**Scottish Index of Multiple Deprivation** ^‡^	2.8	(1.4)	3.0	(1.4)
**PRIOR DISEASE**				
**Type of diabetes mellitus**				
Type 1	8232	(27%)	305	(26%)
Type 2 or other	22886	(74%)	846	(74%)
**Retinopathy grading (worse eye)^¶^**				
No retinopathy (R0)	35	(0%)	9	(1%)
Mild background retinopathy (R1)	29964	(96%)	1126	(98%)
Observable background retinopathy (R2)	1119	(4%)	16	(1%)
**Maculopathy grading (worse eye)^¶^**				
No maculopathy (M0)	26779	(86%)	1032	(90%)
Observable diabetic maculopathy (M1)	4339	(14%)	119	(10%)
**HbA1c (mmol/mol)**	68	(18)	66	(16)
**HbA1c (%)**	8.4	(1.7)	8.2	(1.5)
**eGFR (mL/min/1.73m^2^)**				
<60	4019	(13%)	98	(9%)
≥60	27099	(87%)	1053	(91%)

Results are shown as N (%) or mean (standard deviation)

* based on SCI Diabetes search for summary data regarding patients with (i) non-referable retinopathy at most recent retinal screening, and (ii) eGFR ≥40 mL/min/1.73m^2^ at their most recent blood test;

† ethnicity for potentially eligible individuals based on 26,476 (85% of total) with available information;

‡ based on quintiles of SIMD (calculated based on seven domains: income, employment, education, health, access to services, crime and housing);

¶ worse eye retinopathy defined as R2 > R1 > R0, worse eye maculopathy defined as M1 > M0;

## Novelty Statement

### What is already known?

- Diabetic retinopathy remains a leading cause of visual loss.
- Results from cardiovascular trials of fenofibrate suggest that treatment with the lipid-modifying drug fenofibrate may reduce the progression of diabetic retinopathy. However, no dedicated large-scale trials have yet been conducted to confirm this.

### What this study adds?

- LENS is a streamlined double-masked randomised placebo-controlled trial, embedded within routine care, designed to assess the effect of treatment with fenofibrate in people with early diabetic retinopathy on a composite primary outcome of progression to referable diabetic retinopathy or the need for treatment with intra-vitreal injections, retinal laser or vitrectomy.
