# Supplementary material for: Design, recruitment, and baseline characteristics of the LENS trial
Source: Diabet Med. Author manuscript; Available in PMC 2025 Jul 20. (PMC7617915; doi:10.1111/dme.15310)
Supplement: Supplement [file EMS206871-supplement-Supplement.pdf]

Design, recruitment, and baseline characteristics of the LENS trial

The LENS Collaborative Group

Supplementary Materials

Contents

1. Members of the LENS Collaborative Group (4 pages)

2. Protocol (61 pages)

3. Statistical Analysis Plan (13 pages)

Members of the LENS Collaborative Group

Members are based in the United Kingdom unless otherwise indicated.

Writing Committee: David Preiss*, Jennifer Logue, Emily Sammons*, Mohammed Zayed, Jon Emberson*, Rachel Wade, Karl Wallendszus, Will Stevens, Simon Harding, Graham Leese, Gemma Currie, Jane Armitage*

Writing Committee Affiliations:

Clinical Trial Service Unit and Epidemiological Studies Unit, Nuffield Department of Population Health, University of Oxford, Oxford (*also part of the Medical Research Council Population Health Research Unit at the University of Oxford): D.P., E.S., M.Z., J.E., R.W., K.W., W.S. and J.A.; Faculty of Health and Medicine, University of Lancaster, Lancaster: J.L.; Department of Eye and Vision Science, University of Liverpool and St. Paul's Eye Unit, Liverpool University Hospitals NHS Foundation Trust, Liverpool: S.H.; Molecular and Clinical Medicine, University of Dundee, Dundee: G.L.; School of Cardiovascular & Metabolic Health, University of Glasgow, Glasgow: G.C.

Steering Committee: Paula Williamson (Chairperson, University of Liverpool)*, David Preiss (Chief Investigator, University of Oxford), Jane Armitage (University of Oxford), Jonathan Bodansky (Leeds Teaching Hospitals NHS Trust)*, Allan Cairns (patient representative)*, Sue Dickie (patient representative)*, Jon Emberson (University of Oxford), Gillian Hallard (Royal National Institute of Blind People (RNIB) Scotland)*, Simon Harding (University of Liverpool)*, Gozie Joe Adigwe (previous member, RNIB Scotland)*, Laura Jones (previous member, RNIB Scotland)*, Jennifer Logue (University of Lancaster), Timothy Lyons (Medical University of South Carolina, USA)*, Isla Mackenzie (University of Dundee), Dermot Neely (Newcastle upon Tyne Hospitals NHS Foundation Trust)*, Sarah Wild (University of Edinburgh)

*independent members

Data Monitoring Committee: Ian Young (Chairperson, Queen's University Belfast), Praveen Patel (Moorfields Eye Hospital), Alan Watkins (University of Swansea), Natalie Staplin (Non-voting statistician, University of Oxford)

Central Coordinating Office (University of Oxford):

Central Coordinating Office Management Team: Jane Armitage, Sarah Howard, David Preiss, Emily Sammons, Kate Sawyer, Karen Taylor, Mohammed Zayed; *Project Management*: Sarah Howard, Kate Sawyer, Karen Taylor; *Administration*: Patricia Achiri, Andy Burke, Susan Fathers, Susan Hurley, Gareth McChlery, Kevin Murphy, Sandra Pickworth, Alison Timadjer, Kate Vandenberg, Monique Willett, Liz Wincott; *Call centre*: Matthew Beesley, Charles Cutting, Monica Raj, Ian Roure; *Clinical*: David Preiss, Emily Sammons, Mohammed Zayed, Michelle Goonasekera; *Communications*: Graham Bagley, Anne Whitehouse, Isobel Young; *Contracts*: Gavin Brown; *Data analysis*: Kate Bird, Rosanna Cretney, Heather Halls, Will Stevens, Karl Wallendszus; *IT Validation*: Guanguo Cui, Charles Daniels, Angela Field, Bob Goodenough, Youcef Mostafi, Samee Syed; *Monitoring*: Angela Chamberlain, Carol Knott, Anna Liew, Juliette Meek, Meera Mistry, Andrea Wilson, Patrick Zettegren; *Programming*: Rach AitSadi, Ian Barton, Alex Baxter, Clive Berry, Rosanna Cretney, Rijo Kurien, Michael Lay, Aleksandra Murawska, James Thompson, Allen Young; *Statistics*: Jon Emberson, Enti Spata, Rachel Wade; *Systems Analyst*: Rachel Raff; *Transport*: Crispian Howard

Regional Centre Team (NHS Greater Glasgow and Clyde):

Project Management: Diann Taggart; *Clinical*: Gemma Currie, Paul Connelly, Jennifer Logue

Local Clinical Centres:

NHS Ayrshire and Arran (University Hospital Ayr, University Hospital Crosshouse; 106 participants randomised): Mohan Varikkara (Lead Investigator), Scott Blackwell, Fiona Elliott,

Jacqueline Locke, Angela Sweeney, Stephen Wood; *NHS Borders (Borders General Hospital; 42 participants randomised)*: Olive Herlihy (Lead Investigator), Jeni Darling, Jill Little; *NHS Dumfries and Galloway (Dumfries and Galloway Royal Infirmary; 34 participants randomised)*: Ewan Bell (Lead Investigator), Susan Boytha, Paula Neill; *NHS Fife (Queen Margaret Hospital, Victoria Hospital*; *195 participants randomised*): Caroline Styles (Lead Investigator), Andrew Blaikie, Laura Beveridge, Tina Coventry, Susan Fowler, Karen Gray, Linda Lister, Amanda McGregor, Sandra Pirie, Evgeniya Postovalova*; NHS Forth Valley (Forth Valley Royal Hospital; 97 participants randomised)*: Anna White (Lead Investigator), John Doig (previous Lead Investigator), Stephanie Brogan, Margie Duncan, Angela Gilmour, Lynn Ryan, Anne Todd, Lesley Whitelaw, Elaine Wilson; *NHS Grampian (Aberdeen Royal Infirmary; 93 participants randomised)*: Sam Philip (Lead Investigator), Ann Cadzow, Sharlene Greig, Siby Joseph, Lynne Murray, Julie Toye; *NHS Greater Glasgow and Clyde (Glasgow Royal Infirmary, Queen Elizabeth University Hospital; 108 participants randomised)*: Sonia Zachariah (Local Lead Investigator (joint)), David Carty (Local Lead Investigator (joint)), Lindsey Bailey, Alison Begg, Katherine Brown, Charlotte Clark, Katharine Duffy, Shirley Fawcett, Adam Gill, Emma Hughes, David Jenkins, Laila Martin, Christine McAllister, Mhairi McIntyre, Leeanne Milne, Erin Murphy, Hilary Peddie, Katharine Sharp, Susan Speirs, Joyce Thompson, Lynne Turner; *NHS Highland (Raigmore Hospital; 45 participants randomised)*: Simon Hewick (Local Lead Investigator), Clare Bradley, Jim Finlayson, Laura O'Keeffe; *NHS Lanarkshire (University Hospital Hairmyers, University Hospital Monklands, University Hospital Wishaw; 204 participants randomised)*: Meena Virdi (Local Lead Investigator), Soo Park, Murdina Bell, Maureen Brown, Linda Macliver, Jackie Quigley; *NHS Lothian (Princess Alexandra Eye Pavilion; 80 participants randomised)*: Karen Madill (Local Lead Investigator), Daniel Beck, Freddie Burgess, Karen Charlwood, Lauren Finlayson, Nicola Freeman, Margaret McDonald, Carla Pettifor, Karen Sedstrem, Alice Thomson; *NHS Tayside (Ninewells Hospital; 147 participants randomised)*: Graham Leese (Local Lead Investigator), Ellen Malcolm, Jackie Lindsay, Lisa Stewart

LENS Collaborating Investigators: Jane Armitage (University of Oxford), Helen Colhoun (University of Edinburgh), Gemma Currie (University of Glasgow), Graham Leese (University of Dundee), Robert Lindsay (University of Glasgow), Jennifer Logue (University of Lancaster), John Olson (NHS Grampian), Naveed Sattar (University of Glasgow), Emily Sammons (University of Oxford), Graham Scotland (University of Aberdeen)

Lowering Events in Non-proliferative retinopathy in Scotland (LENS)

A randomised placebo-controlled trial of fenofibrate to prevent progression of non-proliferative retinopathy in diabetes

RESEARCH REFERENCE NUMBERS

IRAS Number:	209579
EudraCT Number:	2016-002656-24
Clinical trials.gov Number:	NCT03439345
ISRCTN Number	ISRCTN15073006
REC Number:	16/WS/0149
Sponsor’s number:	CTSULENS1
NIHR Number:	14/49/84

KEY TRIAL CONTACTS

Chief Investigator	Associate Prof David Preiss<br>MRC Population Health Research Unit<br>Clinical Trial Service Unit, University of Oxford<br>Telephone: +44 (0)1865 743527<br>Email: david.preiss@ndph.ox.ac.uk
Co-investigators	Dr Jennifer Logue (University of Lancaster, UK)<br>Prof Jane Armitage (University of Oxford, UK)<br>Dr John Olson (NHS Grampian, UK)<br>Dr Graham Scotland (University of Aberdeen, UK)<br>Prof Naveed Sattar (University of Glasgow, UK)<br>Dr Graham Leese (University of Dundee, UK)<br>Prof Helen Colhoun (University of Edinburgh, UK)<br>Dr Robert Lindsay (University of Glasgow, UK)<br>Dr Emily Sammons (University of Oxford, UK)<br>Dr Gemma Currie (University of Glasgow, UK)
Trial Managers	Clinical Trial Service Unit, University of Oxford:<br>Sarah Howard<br>Telephone: +44 (0)1865 743825<br>Email: sarah.howard@ndph.ox.ac.uk<br>NHS Greater Glasgow and Clyde:<br>Diann Taggart<br>Telephone: +44 (0)141 314 4407<br>Email: diann.taggart@ggc.scot.nhs.uk
Sponsor	University of Oxford
Funder(s)	National Institute for Health Research
Clinical Trials Unit	Clinical Trial Service Unit and Epidemiological Studies Unit (CTSU), University of Oxford, Richard Doll Building, Old Road Campus, Roosevelt Drive, Oxford, OX3 7LF<br>Telephone: +44 (0)1865 743743; Fax: +44 (0)1865 743985<br>Email: lens@ndph.ox.ac.uk
Statistician	Dr Jonathan Emberson<br>Email: jonathan.emberson@ndph.ox.ac.uk
Trials pharmacy contact	Kevin Murphy<br>Email: kevin.murphy@ndph.ox.ac.uk

TRIAL SUMMARY

Trial Title	A phase 4 randomised placebo-controlled clinical trial of fenofibrate to prevent progression of non-proliferative retinopathy in diabetes
Internal ref. no. (or short title)	LENS: Lowering Events in Non-proliferative retinopathy in Scotland
Clinical Phase	4
Trial Design	Randomised double-blind placebo-controlled trial
Trial Participants	Adult participants with diabetes and observable retinopathy
Planned Sample Size	At least 1,060 participants (approximately 530 on fenofibrate tablets and 530 on placebo tablets)
Follow-up duration	At least 4-years after randomization of the median patient and at least 222 primary outcome events
Planned Trial Period	6 years
Primary research objective	To estimate the effect of allocation to fenofibrate versus placebo on progression to the composite primary outcome of referable diabetic retinopathy (DR) or any of the following treatments for DR: retinal laser therapy, vitrectomy or intra-vitreal injection
Secondary research objectives	To estimate the effect of allocation to fenofibrate versus placebo on:<br>• The composite primary outcome in various subgroups<br>• Individual components of the composite primary outcome<br>• Any progression of DR across the Scottish Diabetic Eye Screening (DES) grading scale<br>• Visual acuity<br>• Hard exudates or blot haemorrhages by the macula<br>• Macular oedema<br>• Visual function and quality of life<br>To evaluate the:<br>• Total cost to the health service (in terms of additional drug treatment and monitoring costs, and health care resource use over follow-up)<br>• Cost-effectiveness (in terms of incremental cost per QALY gained with fenofibrate versus placebo)
Investigational Medicinal Product	Fenofibrate
Formulation, Dose, Route of Administration	One 145mg nanoparticle fenofibrate tablet or matching placebo tablet daily (or one tablet every second day in the context of chronic kidney disease) taken orally

FUNDING AND SUPPORT IN KIND

FUNDERS	FINANCIAL AND NON-FINANCIAL SUPPORT GIVEN
National Institute for Health Research	Financial support
Mylan	Fenofibrate and placebo bulk supplies (free of charge)
CTSU	Cost of packaging, storage of study drug

STUDY SPONSOR AND FUNDER

The LENS trial is sponsored by the University of Oxford. The trial is funded by the National Institute for Health Research. The funder has no influence over trial design, conduct, data analysis and interpretation, manuscript writing, and dissemination of results. The research team will send reports regarding the progress of the trial to the funder at agreed intervals.

CO-ORDINATION OF THE TRIAL

The Central Co-ordinating Office (CCO) for the conduct of the LENS trial is the Clinical Trial Service Unit and Epidemiological Studies Unit (CTSU), University of Oxford. The Regional Co-ordinating Centre (RCC) for the conduct of the trial is based in Glasgow (BHF Glasgow Cardiovascular Research Centre, University of Glasgow together with NHS Greater Glasgow and Clyde). There will be up to 11 NHS Scotland mainland health boards taking part with one or more Local Collaborating Centres (LCC) per health board.

The CCO will have overall responsibility for the trial administration and co-ordination including:

- obtaining Research Ethics Committee (REC), Medicines and Healthcare products Regulatory Agency (MHRA) and Public Benefit and Privacy Panel (PBPP) approval
- training of CCO, RCC and LCC staff
- monitoring of sites and of centrally collected data
- producing abbreviated and detailed patient information leaflets
- providing questionnaires and newsletters, and administering electronic questionnaires
- randomisation service
- the provision of a 24-hr Freephone telephone service for participant and clinical queries
- providing the electronic case report form (eCRF) and trial management system
- producing regulatory reports
- performing statistical data analyses
- securing agreements with NHS health boards to enable insertion of abbreviated trial leaflets into retinal screening results
- maintaining the Trial Master File
- receiving completed abbreviated patient information leaflets and reply slips

The RCC will be responsible for:

- site set-up
- training LCC site staff
- day to day management of sites after set-up
- regular monitoring of participants' renal function using routinely collected blood results via the Scottish Care Information (SCI) Diabetes system, for safety purposes

The LCCs will be responsible for:

- obtaining local NHS Research and Development (R&D) approval (with the assistance of the CCO and RCC)
- contacting potential participants based on Scottish Diabetes Research Network (SDRN) register and DES mail-out and replies to Health Informatics Centre mail out
- determining eligibility during pre-eligibility checks, screening and randomisation visits
- arranging analysis of blood and urine samples for any protocol specified analyses in local NHS laboratories

- transcription of biochemistry results from screening and randomisation visits into eCRF
- randomisation of eligible participants
- seeking information regarding outcomes and other health events of interest from electronic and/or paper health records where relevant
- administering telephone questionnaires
- maintaining site files

The University of Aberdeen will be responsible for health economic analyses.

ROLES AND RESPONSIBILITIES OF TRIAL COMMITEES

TRIAL MANAGEMENT COMMITTEES

- **Trial Management Group (TMG):** the TMG will consist of the LENS investigators, project managers, trial statistician(s), and trial programmer(s). Its primary aim is to deliver the trial in accordance with the protocol. It will meet approximately every 3-6 months, either in person or by teleconference.

- **Local Management Group (LMG):** the LMG will consist of three investigators (David Preiss, Jennifer Logue and Jane Armitage), the project managers and the trial statistician. This group will meet (face-to-face and/or by teleconference) at least monthly and is intended to ensure the smooth operation of the trial on a day-to-day basis. Additional members will be included in specific meetings depending on issues to be dealt with.

- **Trial Steering Committee (TSC):** the TSC will consist of an independent chairperson (a statistician with relevant trial experience), independent physicians with relevant clinical experience, an independent colleague from the Royal National Institute for the Blind (RNIB), at least one patient representative, the Chief Investigator, the trial statistician(s) and other members. In accordance with NIHR guidance, at least 75% of TSC voting members will be independent. Meetings will also be attended by the LENS project managers. The TSC will meet regularly (more often during recruitment), either in person or by teleconference, to discuss trial progress. The TSC will be responsible for protecting patients' interests and provide overall supervision regarding the trial's progress, adherence to the protocol and patient safety. The TSC can decide, based on DMC advice, whether to end or modify the trial or to seek additional data. Unless this occurs, the TSC, the CCO (except those who supply any confidential analyses), the RCC and the LCC will remain ignorant of any interim results until the trial is completed. The TSC is also responsible for decisions regarding the addition of any LENS sub-studies during the conduct of the trial.

- **Data Monitoring Committee (DMC):** This independent committee will consist of three members including two clinicians with relevant experience and an experienced statistician. The DMC will review unblinded analyses of the composite primary outcome, deaths and hospitalisations along with any other analyses requested, according to randomised treatment arm. In the light of these analyses and results from any other trials, the DMC will advise the TSC if, in their view, the randomised comparisons have provided both (a) proof beyond reasonable doubt that for all patients, or for some specific types, fenofibrate therapy is clearly indicated or contra-indicated; and (b) evidence that might reasonably be expected to materially

influence the patient management of many clinicians. The DMC will meet approximately annually with the possibility of additional meetings, and there will be a DMC chairperson's review of the results every 6 months.

Collaborators and all others associated with the trial may write (preferably via the CCO) to the chairman of the DMC at any time to draw attention to any concerns they may have about particular side-effects or about particular categories of patients requiring special consideration, or about any other matters.

LIST OF CONTENTS

Page

RESEARCH REFERENCE NUMBERS 2
KEY TRIAL CONTACTS 3
TRIAL SUMMARY 4
FUNDING AND SUPPORT IN KIND 5
STUDY SPONSOR AND FUNDER 6
CO-ORDINATION OF THE TRIAL 6
ROLES AND RESPONSIBILITIES OF TRIAL COMMITEES 7
Trial Management Committees 7
LIST OF ABBREVIATIONS 11
TRIAL FLOW CHART 13
STUDY PROTOCOL 14
1 BACKGROUND 14
2 RATIONALE 14
3 OBJECTIVES AND OUTCOMES 18
3.1 Objectives 18
3.2 Outcomes 18
3.3 Adjudication of pre-specified outcomes and other events 21
4 TRIAL DESIGN 22
4.1 Study Setting 22
4.2 Eligibility Criteria 22
4.3 Trial Procedures 23
4.4 Recruitment 23
4.5 Screening and Randomisation 25
4.6 Post randomisation follow-up 29
4.7 Trial questionnaires 32
4.8 Stopping study treatment, change of consent and withdrawal 33
4.9 Analysis of blood and urine samples 34
4.10 End of trial 34
4.11 Trial Medication 35
5 PHARMACOVIGILANCE 40
5.1 Definitions to be used in lens 40
5.2 Events associated with fenofibrate use 40
5.3 Recording and reporting adverse events 41
5.4 Notification of specific events 43
5.5 Responsibilities 44
6 STATISTICS AND DATA ANALYSIS 45
6.1 Sample size calculation 45

6.2 Statistical analysis 47
7 DATA HANDLING 49
7.1 Source Data 49
7.2 Access to Data 49
7.3 Data protection and patient confidentiality 50
7.4 Access to the final trial dataset 50
7.5 Archiving 50
8 MONITORING, AUDIT & INSPECTION 50
9 ETHICAL AND REGULATORY REPORTS AND CONSIDERATIONS 51
9.1 Peer review 51
9.2 Public and Patient Involvement 51
9.3 Amendments 52
9.4 compliance 52
9.5 Financial and other competing interests 52
9.6 Indemnity 52
10 DISSEMINATION POLICY 53
11 REFERENCES 54
12 APPENDICES 56
12.1 Appendix 1 - Risk assessment 56
12.2 Appendix 2 – Authorisation of Local collaborating Centres 57
12.3 Appendix 3 – Schedule of Assessments 58
12.4 Appendix 4 – Protocol Amendment History 59

LIST OF ABBREVIATIONS

ACCORD	Action to Control Cardiovascular Risk in Diabetes
AE	Adverse Event
ALT	Alanine aminotransferase
AR	Adverse Reaction
AST	Alanine aminotransferase
BNF	British National Formulary
CCO	Central Co-ordinating Office
CHI	Community Health Index
CI	Chief Investigator
CK	Creatine kinase
CTA	Clinical Trial Authorisation
CTSU	Clinical Trial Service Unit and Epidemiological Studies Unit
DM	Diabetes Mellitus
DMC	Data Monitoring Committee
DR	Diabetic Retinopathy
DES	Diabetic Eye Screening
DSUR	Development Safety Update Report
eCRF	Electronic Case Report Form
eDRIS	Electronic Data Research and Innovation Service
eGFR	Estimated Glomerular Filtration Rate
ETDRS	Early Treatment Diabetic Retinopathy Study
EU	European Union
EudraCT	European Clinical Trials Database
FIELD	Fenofibrate Intervention and Event Lowering in Diabetes
GCP	Good Clinical Practice
GMP	Good Manufacturing Practice
GP	General Practitioners
HIC	Health Informatics Centre
ICF	Informed Consent Form
IMP	Investigational Medicinal Product
ISD	Information Services Division
ISF	Investigator Site File
LENS	Lowering Events in Non-proliferative retinopathy in Scotland
LMG	Local Management Group
LFT	Liver function tests
LCC	Local Collaborating Centre
MHRA	Medicines and Healthcare products Regulatory Agency
NHS	National Health Service
NIHR	National Institute for Health Research
NRS	National Records of Scotland
NSAID	Non-steroidal anti-inflammatory drug
OCT	Optical coherence tomography
OPCS	Office of Population Censuses and Surveys
PBPP	Public Benefit and Privacy Panel
PI	Principal Investigator

PIL	Participant Information Leaflet
QALY	Quality adjusted life-years
QP	Qualified Person
RCC	Regional Co-ordinating Centre
RCT	Randomised Control Trial
REC	Research Ethics Committee
RNIB	Royal National Institute for the Blind
SAE	Serious Adverse Event
SCI	Scottish Care Information
SDRN	Scottish Diabetes Research Network
SOP	Standard Operating Procedure
SmPC	Summary of Product Characteristics
SMR	Scottish Morbidity Record
SSAR	Suspected Serious Adverse Reaction
SUSAR	Suspected Unexpected Serious Adverse Reaction
TMG	Trial Management Group
TMS	Trial Management System
TSC	Trial Steering Committee
TMF	Trial Master File
ULN	Upper limit of normal
VFQ-25	Visual Function Questionnaire-25

TRIAL FLOW CHART

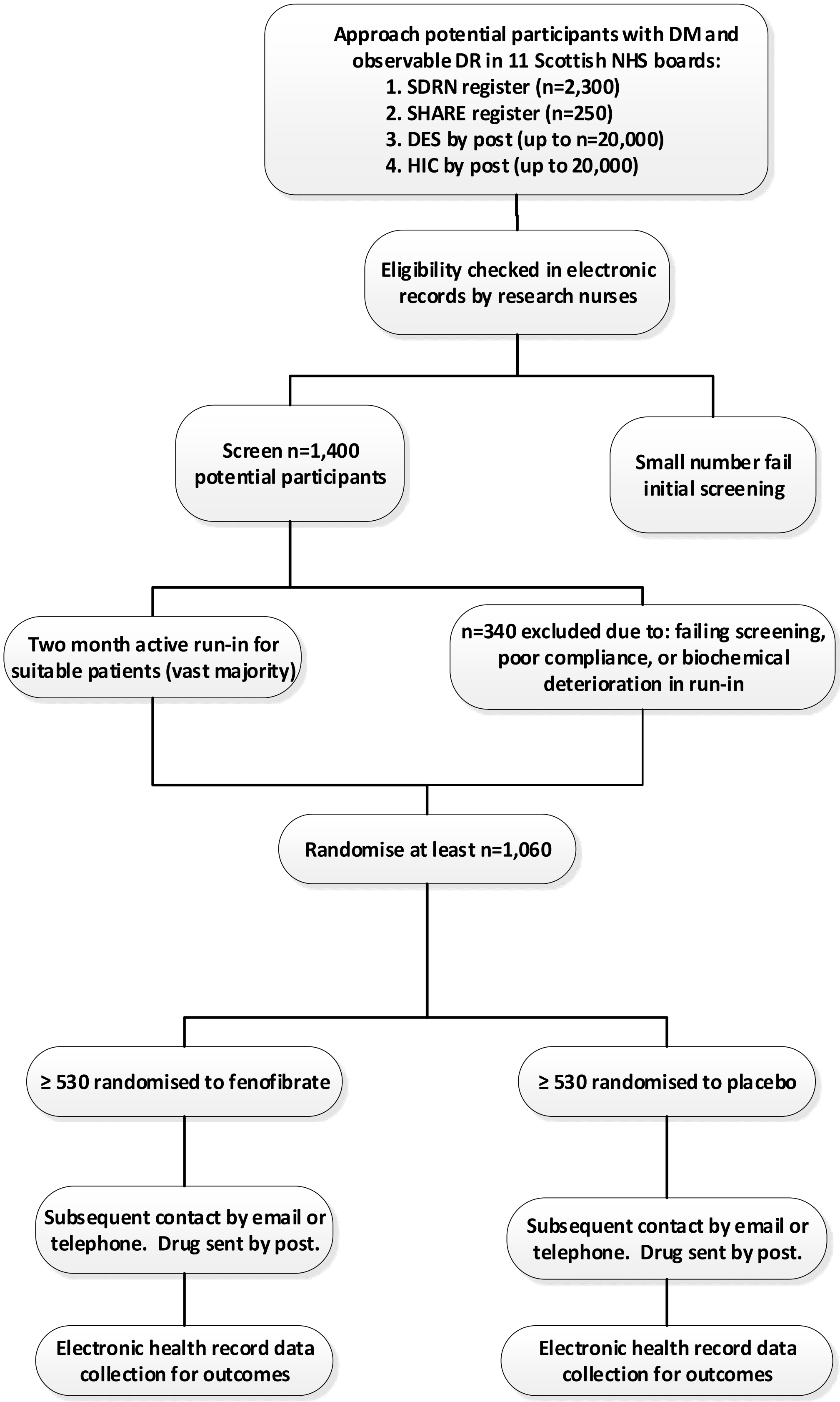

STUDY PROTOCOL

LENS: Lowering Events in Non-proliferative retinopathy in Scotland

1 BACKGROUND

Diabetic retinopathy (DR) and diabetic maculopathy are common microvascular complications in patients with diabetes mellitus (DM)[1]. DR is a progressive disease that includes the following stages: no apparent DR, non-proliferative DR, and proliferative DR. Non-proliferative DR is characterised by the presence of observable changes in the retina (venous dilatation, microaneurysms, retinal haemorrhages, retinal oedema, and hard exudates). Proliferative DR is characterised by neovascularisation which occurs in response to the hypoxic condition of the retina. The development of these abnormal new retinal blood vessels may lead to vitreal haemorrhage, retinal detachment or neovascular glaucoma. Diabetic maculopathy is characterised by the development of hard exudates or blot haemorrhages close to the fovea. Maculopathy carries the risk of retinal thickening at the macula which is a major threat to vision.

Diabetic retinopathy and maculopathy (referred to in the protocol hereafter only as diabetic retinopathy [DR]) remains a leading causes of blindness and poor vision[2], one of the most feared complications of DM. In Scotland, Diabetic Eye Screening (DES) Collaborative data show that in diabetic individuals not already attending specialist eye clinics, more than 30% have DR in one or both eyes. More than 5,500 diabetic patients annually develop referable DR in Scotland[3]. Of those with observable DR (defined as R2 or M1 or bilateral R1 on the Scottish DES grading system – see Table 1), DES data indicate that 29% develop referable disease within 4 years. DR leads to the certification of blindness in about 1,400 patients in the UK each year[2,4] making it one of the most important causes of blindness in people of working age. In a survey conducted in a randomly selected group of 586 patients with DM from Tayside, Scotland, the presence and intensity of fear of visual loss was explored[5]. Fear of visual loss was frequent for 37% and intense for 47%. Notably these patients were not selected based on having any DR. Patients with clinically significant DR demonstrated even higher levels of concern for loss of vision.

While blindness is the most obvious concern raised by patients, the potential impact of reduced vision on quality of life extends well beyond this. Qualitative studies have been conducted in adults with DM and mild, moderate or severe non-proliferative DR or proliferative DR[6]. Participants described a range of problems such as difficulty driving (especially at night) and reading, notably at all grades of DR. Those with more severe eye disease forego important life aspects like working and activities related to DM treatment can become difficult, e.g. reading food labels, taking insulin injections, glucose testing. Loss of independence is a major fear, especially mobility and increased fear of accidents. It is in the interests of the National Health Service (NHS) for patients to remain independent, and preservation of vision is an important goal in DM.

2 RATIONALE

While the risk of cardiovascular events in DM has been substantially reduced by various preventive strategies, there are few effective options to slow the progression of microvascular disease. Historical studies like the UK Prospective Diabetes Study[7] and the Diabetes Control and Complications Trial[8] showed that moderately good glycaemic control could reduce microvascular complications compared with poor control. However, recent trials of intensive glucose-control, in which glycaemic levels in the control arms were considerably better than in the historical trials and

therefore more relevant to contemporary care, showed no benefit for microvascular outcomes like DR and nephropathy[9]. We are, therefore, left with few effective options apart from screening for progressive retinal disease and then using interventions like laser therapy, intravitreal anti-vascular endothelial growth factor injection and vitrectomy at a late stage when DR has already progressed to a clinically significant grade.

Clofibrate, the precursor of fenofibrate, was studied in the 1960-1970s. Although the drug was discontinued due to the increased risk of gallbladder complications, various small trials showed that clofibrate could reduce hard exudates in patients with DM[10]. Fenofibrate is a lipid-modifying agent which activates peroxisome proliferator-activated receptor alpha to lower circulating triglycerides[11]. It has modest HDL-cholesterol raising and LDL-cholesterol lowering effects. Two major cardiovascular trials of fenofibrate therapy conducted in type 2 DM patients have included DR as a tertiary outcome[12],[13]. Both suggested that fenofibrate may provide microvascular benefit. The Fenofibrate Intervention and Event Lowering in Diabetes (FIELD) study was a placebo-controlled clinical trial conducted in 9795 participants with type 2 DM who were not on a statin to assess the effect of fenofibrate on cardiovascular outcomes[12]. In FIELD, 1012 participants agreed to take part in the DR sub-study with retinal photographs at 0, 2 and 5 years. Allocation to fenofibrate was associated with a reduction in the composite DR endpoint comprising the need for retinal laser therapy, the development of macular oedema or 2-step Early Treatment Diabetic Retinopathy Study (ETDRS) grade progression[14]. Based on 53 (11.1%) vs. 75 (16.1%) events the reduction was 34% (95 CI 6-53%; p=0.022) over 5 years of follow-up. In the full FIELD cohort (n=9795), self-reported laser therapy was also reduced by 31% (16%-44%); p=0.0002 based on 164 (3.4%) vs. 238 (4.9%) reports. The placebo-controlled Action to Control Cardiovascular Risk in Diabetes (ACCORD)-Lipid study was designed to test the effect of fenofibrate on cardiovascular outcomes in 5518 participants with type 2 DM who were all on open-label simvastatin therapy [13]. In ACCORD-Lipid, 1593 participants took part in the Eye sub-study which included two standardised eye examinations with fundal photography at 0 and 4 years. The composite outcome of laser treatment, vitrectomy or 3-step progression on the ETDRS scale was reduced by 40% (13-58%; p=0.006; 52 [6.5%] vs. 80 [10.2%] events)[15]. Various other DR outcomes were not improved by fenofibrate therapy however. Recent work in an animal model of DR suggests that fenofibrate may be acting directly within the eye and that any effect on circulating lipids may therefore not be relevant to its potential effects on microvascular disease[16].

Criticisms of these cardiovascular trials' retinal findings are:

i) the majority of participants had no DR at baseline, a group unlikely to be treated routinely with fenofibrate
ii) the use of composite or self-reported outcomes which may be less robust than objectively reported results
iii) that eye outcomes were tertiary outcomes rather than the primary outcome of the trial
iv) that the trials were conducted in participants with lipids and HbA1c within certain pre-specified limits and may therefore not be generally applicable to other patients with DM, including those with type 1 DM

Notably a recent Primary Care Diabetes Society survey indicated that 76% of general practitioners (GPs) who are aware of the DR findings from FIELD and ACCORD were unlikely to use fenofibrate to treat DR without further trial evidence. Guidelines are also unclear regarding the use of fenofibrate for DR. The Royal College of Ophthalmologists suggests its ‘consideration’ in non-proliferative DR (grade B evidence)[17]. DM guidelines from the National Institute for Health and Care Excellence (NICE) and the Scottish Intercollegiate Guidelines Network do not mention

fenofibrate[18,19]. Australia recently approved fenofibrate for DR. Trial registries confirm that only one fenofibrate trial is currently being considered or conducted, namely FAME-1-EYE[20]. However, this is a trial restricted to patients with type 1 DM and established macular thickening.

There is a pressing need for better evidence for the effect of fenofibrate on the progression of DR from a dedicated large randomised controlled trial (RCT). Given the nature of DR, a slowly progressive condition, it is necessary to recruit individuals at relatively high risk of the condition progressing, and to treat and follow them for a number of years for a RCT to have sufficient power to detect plausible effects. We have designed a double-blinded randomised placebo-controlled trial, LENS. LENS is designed to achieve these objectives at low cost, making use of existing sources of relevant healthcare data to both recruit and follow participants. Scotland is a leader in terms of such healthcare repositories, central to which is the use of each person's Community Health Index (CHI) number. This provides the excellent environment to conduct such a trial.

LENS will recruit and randomise at least 1,060 patients with diabetes and retinopathy to receive either fenofibrate or placebo tablets, to be taken orally, for at least a median of 4 years (counted as 4 years from randomization of the median participant). Prior to randomisation, all participants who are eligible at screening will enter an active run-in phase in which they will be treated with open-label fenofibrate tablets for approximately 8 weeks. The purposes of the active run-in are to:

i) maximise adherence during the trial, thereby providing robust results, and
ii) allow the research team to establish the best dosing regimen for each participant during the trial.

The dose of fenofibrate tablets to be used in LENS is 145mg. Each participant's dosing regimen will be determined by their renal function. Those with normal renal function will take one fenofibrate tablet daily during the active run-in and one study tablet (fenofibrate or placebo) daily after randomisation, whereas participants with chronic kidney disease will take one fenofibrate every second day during the active run-in and one study tablet every second day after randomisation.

Table 1. Scottish DES Collaborative grading system for DR

Grading	Description	Outcome
**RETINOPATHY (excluding the macula)**		
R0	*No DR anywhere*	Rescreen in 12-24 months
R1*	*Background DR – mild*<br>The presence of at least one of any of the following features anywhere<br>• dot haemorrhages<br>• microaneurysms<br>• hard exudates<br>• cotton wool spots<br>• blot haemorrhages§<br>• superficial/ flame shaped haemorrhages	Rescreen in 12 months
R2*	*Background DR – observable*<br>Four or more blot haemorrhages§ in one hemi-field only (Inferior and superior hemi-fields delineated by a line passing through the centre of the fovea and optic disc)	Rescreen in 6 months
R3	*Background DR – referable*<br>Any of the following features:<br>• Four or more blot haemorrhages§ in both inferior and superior hemi-fields<br>• Venous beading<br>• IRMA	Refer to ophthalmology
R4	*Proliferative DR*<br>Any of the following features:<br>• Active new vessels<br>• Vitreous haemorrhage	Refer to ophthalmology
R6	*Not adequately visualised*<br>Retina not sufficiently visible for assessment	Technical failure
**MACULOPATHY**		
M0	*No maculopathy*<br>No features ≤2 disc diameters from the centre of the fovea sufficient to qualify for M1 or M2	Rescreen in 12-24 months
M1*	*Observable maculopathy*<br>Lesions as specified below within a radius of >1 but ≤2 disc diameters the centre of the fovea:<br>• Any hard exudates	Rescreen in 6-12 months
M2†	*Referable maculopathy*<br>Lesions as specified below within a radius of ≤1 disc diameter of the centre of the fovea:<br>• Any blot haemorrhages§<br>• Any hard exudates	May require referral to ophthalmology

* **Definition of observable DR in LENS**: R1 in both eyes and/or R2 in at least one eye at the most recent retinal screening visit; and/or M1 in at least one eye at any retinal screening in the last 3 years (and R3, R4 or M2 [i.e. referable DR] not present in either eye)

§ blot haemorrhage has same or greater diameter as retinal vein crossing the optic disc

† At the start of LENS in 2018 and until end-2021, all M2 led to specialist ophthalmology referral. During 2022, DES started to introduce a phased change to the management pathway of patients with M2 disease. In the new pathway, M2 in the context of poor visual acuity (e.g. 6/9.5 or worse) leads to optical coherence tomography (OCT) imaging. If OCT shows macular oedema, the patient is referred to an ophthalmologist. Patients who do not require OCT, or in whom there is no evidence of macular oedema on OCT, are not automatically referred. Given that M2 (or equivalent disease) requires specialist referral/review in many countries, the LENS protocol will continue to categorise M2 as referable disease.

3 OBJECTIVES AND OUTCOMES

The main hypothesis is that fenofibrate therapy will slow the progression of observable DR to referable DR or DR requiring laser or surgical treatment when compared with placebo. Objectives and outcomes are summarised in Table 2.

3.1 OBJECTIVES

3.1.1 Primary objective

The primary objective is to compare the effect of allocation to fenofibrate versus placebo on the composite of progression of observable DR to referable DR, or any of retinal laser therapy, vitrectomy or intra-vitreal injection of medication due to DR.

3.1.2 Secondary objectives

Secondary objectives are to compare the effect of allocation to fenofibrate versus placebo on:

(i) the occurrence of the composite primary outcome in various subgroups
(ii) the individual components of the composite primary outcome (namely progression of DR to referable DR; and retinal laser therapy, vitrectomy or intra-vitreal injection for DR)
(iii) any progression of DR across the DES grading scale
(iv) visual acuity
(v) the presence of hard exudates or blot haemorrhages (M2) within 1 disc diameter of the macula
(vi) macular oedema
(vii) visual function
(viii) quality of life
(ix) total cost to the health service
(x) cost-effectiveness

3.2 OUTCOMES

3.2.1 Primary outcome

The primary outcome is the composite of progression from observable DR to referable DR, or any of retinal laser therapy, vitrectomy or intra-vitreal injection of medication due to DR. Referable DR is defined according to the Scottish DES grading as R3 or R4 or M2 in at least one eye on the DES grading scheme (see Table 1).

For those participants in whom the composite primary outcome is reported (whether via data linkage or questionnaire), LCC research nurses will review their electronic health records periodically to determine any therapy, i.e. retinal laser, vitrectomy or intra-vitreal injection, which was provided. While most primary outcomes are expected to come from routine retinal imaging, any findings of referable DR recorded during clinical examination (e.g. by an ophthalmologist) will also be included as an outcome.

3.2.2 Secondary outcomes

The following are pre-specified secondary outcomes:

- The individual components of the composite primary outcome, i.e.:
  - Progression of DR to referable DR
  - Retinal laser therapy for DR
  - Vitrectomy for DR

- Intra-vitreal injection for DR

- Composite of retinal laser, vitrectomy or intra-vitreal injection for DR
- Any progression of DR across the DES grading scale
- Visual acuity (according to LogMAR or Snellen chart measurement conducted at DES visits)
- The development of hard exudates or blot haemorrhages within 1 disc diameter of the macula (defined as M2 in the DES grading scheme; see Table 1)
- The development of macular oedema*
- Visual function (according to the Visual Function Questionnaire [VFQ]-25 questionnaire)
- Quality of life (according to the EQ-5D questionnaire)
- Total cost to the health service (in terms of additional drug treatment and monitoring costs, and health care resource use over follow-up)
- Cost-effectiveness (in terms of incremental cost per quality adjusted life-years [QALY] gained with fenofibrate versus placebo)

*During 2022, Scotland started to introduce optical coherence tomography (OCT) imaging for certain patients who reach M2 grading (based on visual acuity). The availability of data from OCT imaging is yet to be established. The presence of macular oedema (i.e. intra-retinal fluid) has therefore been included as an additional secondary outcome.

3.2.3 Tertiary outcomes

Tertiary outcomes include: urine albumin:creatinine ratio, the occurrence of major cardiovascular events (myocardial infarction, stroke, coronary and peripheral revascularisation), and minor and major non-traumatic lower limb amputation (minor [defined as distal to the ankle] or major [defined as through or proximal to the ankle]).

3.2.4 Longer term follow up

Consent will be sought from participants at baseline to facilitate investigation of the longer term effects of ~4 years' fenofibrate treatment after the main study ends. Long-term outcomes may include the development of significantly impaired vision or blindness, DR related eye procedures, cardiovascular outcomes, progression to end-stage renal failure and other outcomes. Any long term follow-up will be described in a further protocol and new ethical and Public Benefit and Privacy Panel (PBPP) approvals will be required before such work is undertaken (see section 4.6.5).

Where possible, we will seek to meta-analyse LENS results with results from FIELD and ACCORD and other trials of fenofibrate therapy.

Table 2. Summary of LENS objectives and outcomes

Objectives	Outcome Measures	Time-point(s) of evaluation
**Primary Objective:** to compare the effect of allocation to fenofibrate versus placebo on the primary outcome	**Primary outcome:** the composite of progression from observable DR to referable DR (defined according to the Scottish DES grading as R3 or R4 or M2 in at least one eye*), or any of retinal laser therapy, vitrectomy or intra-vitreal injection of medication due to DR, whichever occurs first.<br><br>* from retinal imaging or clinical examination	• 6-12 monthly retinal screening;<br>• 6 monthly trial-specific questionnaires
**Secondary Objectives:** to compare the effect of allocation to fenofibrate versus placebo on the secondary outcomes	**Secondary outcomes:**<br>• the occurrence of the composite primary outcome in various subgroups<br>• the individual components of the composite primary outcome (namely progression of DR to referable DR; and retinal laser therapy, vitrectomy or intra-vitreal injection for DR);<br>• Any progression of DR across the DES grading scale;<br>• Visual acuity on LogMAR or Snellen scale<br>• the presence of hard exudates or blot haemorrhages (M2) within 1 disc diameter of the macula<br>• the presence of macular oedema<br>• Visual function<br>• Quality of life<br>• Total cost to the health service and cost-effectiveness.	• As for the primary outcome<br>• As for the primary outcome<br>• 6-12 monthly retinal screening<br>• 6-12 monthly retinal screening<br>• 6-12 monthly retinal screening<br>• OCT analysis<br>• VFQ-25 questionnaire at 2 years and trial end<br>• EQ-5D questionnaire at 2 years and trial end<br>• Combination of data sources
**Tertiary Objectives:** to compare the effect of allocation to fenofibrate versus placebo on the tertiary outcomes	**Tertiary outcomes:**<br>• urine albumin:creatinine ratio<br>• the occurrence of major cardiovascular events (myocardial infarction, stroke, coronary and peripheral revascularisation)<br>• minor and major non-traumatic lower limb amputation (minor [defined as distal to the ankle] or major [defined as through or proximal to the ankle])	• Routinely collected biochemistry via SCI-Diabetes<br>• Linkage to SMR01 records<br>• Linkage to SMR01 records and 6 monthly questionnaires

Where retinopathy and maculopathy gradings of retinal images contribute to pre-specified outcomes, the grading results generated by the NHS Scotland Diabetic Eye Screening program will be used for the pre-specified analyses. However, it is also intended for University of Oxford investigators to use image analysis software for exploratory analyses of the effect of fenofibrate on DR (after the main trial is completed) and for observational analyses to better understand drivers of DR progression.

3.3 ADJUDICATION OF PRE-SPECIFIED OUTCOMES AND OTHER EVENTS

Primary, secondary and tertiary outcomes will not be adjudicated with the exception of any medical eye procedures, any report of vitreous haemorrhage and the development of macular oedema. Any such adjudication will be conducted by experienced doctors at the CCO who are blinded to treatment allocation. To inform adjudication LCCs will collect supporting documentation e.g. hospital discharge or surgical operation report or OCT report and submit to the CCO for review. Documentation of SSARs will be sought to confirm or refute the reported diagnosis.

LCCs will be required to verify any cardiovascular events, lower limb amputations and deaths which are reported to the CCO by questionnaire or other communication from any participant or clinician. Hospitalisations for non-cardiovascular reasons will be tabulated but not verified by LCCs.

Events which are reported and are unrefuted will be assumed to have occurred regardless of the source of information (e.g. health records or participant or GP). Where possible, comparison will be made between different registry sources of the same data for verification purposes.

4 TRIAL DESIGN

LENS is a multicentre phase 4 randomised placebo-controlled clinical trial.

4.1 STUDY SETTING

The national DES service provides regular retinal imaging and grading for all 250,000 with DM in Scotland. Retinal screening appointments are organised by each health board. Patients receive a summary of their results by post a few weeks after their visit. The trial will be conducted in this real-life setting.

LENS is a multicentre trial to be conducted in up to 11 mainland Scottish NHS Health boards. Each Health board will have at least one LCC and it is anticipated that the majority of Health boards will have one LCC. The trial will be conducted using a pragmatic streamlined trial design with the only planned face-to-face visits at the LCC being an initial screening visit, followed by a randomisation visit eight weeks later. The 24-hour Freephone service (**0808 164 5090**) established by the Clinical Trial Service Unit and Epidemiological Studies Unit (CTSU), University of Oxford, will allow study participants and clinical staff involved in their care to discuss any aspects of the trial with experienced clinical staff, thereby helping to ensure good compliance and the early identification of any major problems.

Participants will be recruited either via direct contact (if the person has previously given permission to do so by agreeing to join a research registry) or via postal invitation (under which circumstances the research team will have no knowledge of identifiable data for any individual until that individual returns a reply slip) which will either be included with their NHS DES screening results or mailed to potential participants by the Health Informatics Centre (University of Dundee) Safe Haven. The majority of participants are likely to be recruited via postal invitation. Participants will be recruited regardless of whether their DM is treated in primary or secondary care. LCCs will typically, though not necessarily, be at major hospitals.

The pathway of standard care will not be altered for LENS participants. They will continue to be followed up according to standard NHS practice. The DES grading system, which operates to NHS Quality Improvement Scotland standards and applies strict external quality control procedures, will have no knowledge of which retinal images belong to LENS participants. If a participant's DR grading deteriorates to a level where specialist review and intervention is required, this will occur as normal.

4.2 ELIGIBILITY CRITERIA

4.2.1 Inclusion criteria

- Subjects capable of giving informed consent
- DM (any type except gestational diabetes)
- Age ≥18 years
- Observable DR (defined as R1 in both eyes or R2 in one/both eyes at the most recent retinal screening assessment; or M1 in one/both eyes at any retinal screening assessment in the 3 years prior to screening)
- Willing to either complete electronic questionnaires or conduct telephone interviews for collection of questionnaire data once every 6 months

4.2.2 Exclusion criteria

- Referable DR (defined as R3 or R4 or M2 in one or both eyes)

- History of gallbladder disease (cholecystitis, symptomatic gallstones, cholecystectomy)
- History of acute or chronic pancreatitis
- Alanine aminotransferase (ALT) or aspartate aminotransferase (AST) >2X the upper limit of normal (ULN) according to local NHS laboratory reference range at screening visit
- ALT or AST >2.5X ULN according to local NHS laboratory reference range at randomisation visit
- Creatine kinase (CK) >3X ULN according to local NHS laboratory reference range at screening visit
- CK >3X ULN according to local NHS laboratory reference range at randomisation visit
- Estimated glomerular filtration rate (eGFR) <40mL/min/1.73m^2 at screening visit
- eGFR <30mL/min/1.73m^2 at randomisation visit
- Cirrhosis of any aetiology or any other serious hepatic disease (investigator opinion)
- Female who is pregnant, breastfeeding, currently trying to become pregnant, or of child-bearing potential and not practising birth control (as defined in section 4.11.12)
- Ongoing vitamin K antagonist (warfarin, phenindione, acenocoumarol), cyclosporine, colchicine, ketoprofen, daptomycin, fibrate therapy, or treatment with rosuvastatin 40mg daily
- Previous myositis, myopathy or rhabdomyolysis of any cause, or diagnosed hereditary muscle disorder
- Ongoing renal replacement therapy
- Any previous organ transplant
- Previous reported intolerance to any fibrate
- Medical history that might limit the individual's ability to take trial treatments for the duration of the study (e.g. severe respiratory disease, history of cancer within last 5 years other than non-melanoma skin cancer; or recent history of alcohol or substance misuse)
- Any other significant disease or disorder which, in the opinion of the Investigator, may either put the participant at risk because of participation in the trial, or may influence the result of the trial, or the participant's ability to participate in the trial
- LENS participants can participate in other research studies, including clinical trials. The only exclusions related to co-enrolment will be: if any other study or trial excludes co-enrolment or if the intervention being investigated in another trial has the potential to interact with fenofibrate therapy.
- Not adherent to active run-in treatment

Any queries regarding eligibility due to participation in other trials will be addressed by experienced study doctors at the CCO.

4.3 TRIAL PROCEDURES

The Schedule of Assessments is provided in Appendix 3.

4.4 RECRUITMENT

4.4.1 Patient identification

The experience of the CCO and Regional Co-ordinating Centre based on large trials like ASCEND[21] and similar smaller mail-out studies like CAMERA[22], suggests that such approaches typically lead to the enrolment of 4-5% of recipients. This necessitates mailing information to a large number of potential participants. Potential participants will be approached using the following methods:

- via the patient research register of the Scottish Diabetes Research Network (SDRN)[23]
- via mail-out with DES results
- via mail-out by the Health Informatics Centre (HIC; University of Dundee) Safe Haven's patient recruitment service (https://www.dundee.ac.uk/hic/patientrecruitment/)
- via the SHARE register[24]

GPs responsible for the care of consenting and eligible patients will be informed by letter from the CCO that their patient wishes to take part. GPs will be able to discuss this with the research team and withdraw the patient if they consider there is any reason not to randomise that patient. Repeat pre-screening (i.e. assessment of eligibility prior to the screening visit) will be permitted for interested patients who are initially found to be ineligible. There will be a trial website (https://www.ctsu.ox.ac.uk/lens) to provide information about the study for individuals who may be interested in participating as well as those who are active participants, for example; the participant information sheet and participant bulletins and newsletters.

4.4.2 Recruitment via the SDRN register

SDRN holds a national register of patients with DM in Scotland who have given consent to be approached regarding research studies[23]. SDRN will provide lists of potentially eligible participants to LCCs in each health board who have agreed to be contacted by phone. The LCC research nurse will phone each patient on the list and (i) ask if they would be interested in receiving further information regarding the LENS trial and (ii) seek verbal consent to check key eligibility criteria in their health records (specifically their most recent eGFR result and most recent retinal screening result). If they agree, they will then be mailed a detailed patient information leaflet (PIL), followed by a further telephone call from the LCC research nurse to discuss the trial, review contact details and arrange a Screening visit if the trial is of interest. Due to the nature of the SDRN register, patients are not uniformly distributed across Scotland. Consequently, this is likely to be an efficient method of recruitment in some health boards but not in others. In July 2014, the total number of patients on the SDRN register likely to be suitable for LENS (based on DES grading) was approximately 2,000.

4.4.3 Recruitment via DES mail-out

Based on DES data, it is estimated that at least 25,000 patients with DM have the appropriate DR grading to be suitable for LENS. Of these, at least 20,000 regularly attend retinal screening via DES. DES will assist with recruitment in LENS. A few weeks after a patient's retinal screening visit, it is standard NHS practice for patients to receive a letter (sent from their NHS health board) with a description of their DR results. The research team will liaise with relevant contacts in each health board to ensure that an abbreviated PIL (with FREEPOST reply slip) regarding LENS is enclosed with the results of their routine retinal screening visit. The content of the results letter enables this to be done relatively selectively, so those patients with grades of DR who may be eligible for LENS will receive the abbreviated trial leaflet. Interested patients can complete the reply slip with their contact details and return it via FREEPOST. It will be specified that, by completing and returning the reply slip, a patient is giving permission for the LCC research nurse or LCC study doctor to check their eligibility for the trial.

The research team will use the returned reply slips to populate a list of potential participants with the information provided. This will allow LCC research nurses to perform preliminary eligibility checks and follow-up with a telephone call to discuss the trial, and check and update contact information, complete the preliminary eligibility checks and arrange local appointments for screening visits. The LCC research nurse will also mail a copy of the detailed PIL to patients for whom screening visits are arranged. As eligible patients have retinal screening performed every 6-

12 months it enables the research team to contact large numbers of potential participants at the time that they receive their retinal screening results. Where it is clear that an individual who has returned a reply slip will not be eligible, a letter of appreciation will be mailed to the relevant patient.

It is possible that during recruitment for LENS, DES will change its procedures for sending appointments and results from the current mail-based system to a web-based electronic system for a subgroup of individuals who agree to such a change. Should this occur, the process of invitation will be amended in conjunction with DES to allow sending of electronic versions of the abbreviated trial leaflet to such patients, or downloading of the leaflet, requesting that participants reply to the CCO by email.

4.4.4 Recruitment via HIC mail-out

On behalf of each health board, SCI Diabetes will create lists of potential participants using the LENS Trial inclusion criteria, i.e. age, DES retinopathy grading and renal function. Lists generated will be securely transferred to the relevant health board for review and onward secure transfer to the HIC's recruitment service. (For some boards such as NHS Tayside, HIC already holds SCI-diabetes data locally on their NHS servers, negating the need a file transfer between SCI-diabetes, the boards and HIC). HIC's recruitment service will mail information regarding LENS (in batches) to potentially eligible participants across mainland Scotland. These mail-outs will include a named invitation letter (signed by the lead DES clinician for that patient's health board), a reply slip, a FREEPOST envelope and a detailed PIL. The return of a reply slip by a potential participant will allow LCC research nurses to perform preliminary eligibility checks and follow-up with a telephone call to discuss the trial. The LCC research nurse will then check and update contact information, complete the preliminary eligibility checks and arrange local appointments for screening visits. Where it is clear that an individual who has returned a reply slip will not be eligible, a letter of appreciation will be mailed to the relevant patient. HIC will send reminder invitation letters to individuals who don't respond to the initial mailing.

As with recruitment via DES mail-out, the research team will have no knowledge of any potential participant's details unless that individual gives permission to contact them by returning a reply slip by FREEPOST. This method of recruitment has been supported by the DES lead clinicians in whose name HIC will mail out the invitations (see letters of support), and it will require national PBPP approval. This national strategy for recruitment of patients with diabetes across Scotland has recently been approved for the adAPT trial (http://adaptdiabetes.org/in-brief-2/).

4.4.5 Recruitment via the SHARE register

SHARE is a recently introduced program in Scotland to which individuals give permission to be contacted regarding studies for which they may be eligible[24]. It is unclear how many of the patients in the SHARE database might be suitable for the trial, and how much overlap there may be with the SDRN register. Never-the-less the option to use the register as a source for recruitment will be kept open. The same process will be followed as is planned for the SDRN register.

4.5 SCREENING AND RANDOMISATION

4.5.1 Screening visit

Potential participants will be asked to attend an appointment at the LCC where they will be assessed by a research nurse. By the time of the screening visit, these individuals will already have had time to consider participation based on information from a detailed telephone discussion with the local research nurse and information in the detailed PIL. At the visit, the research nurse and

potential participant will discuss the trial in detail. Data will be entered directly into the eCRF with limited use of paper wherever possible. After welcoming the patient, relevant details (name, contact details, details of patient's GP and GP practice, date of birth, sex, CHI number) will be entered (if not yet recorded) or checked (if already recorded) in the Trial Management System (TMS). Eligibility criteria and other relevant clinical information (history of eye disease or procedures, previous cardiovascular events, date of DM diagnosis and contraindicated medications, smoking history, other medical history) will be recorded using available clinical data (using the NHS board's clinical portal and SCI Diabetes[25]) and from a clinical history. Patients will be asked, if willing, to provide details of a friend or relative living at a different address who may be contacted in the event of loss of contact.

Informed consent will be obtained before any research procedures are conducted (see below). After informed consent has been obtained, blood and urine samples will be taken to check biochemistry:

- renal function
- liver function tests – LFT (specific tests in a LFT profile will differ between health boards, which is acceptable as long as at least one of ALT or AST is measured)
- CK
- HbA1c
- random lipid profile, including at least total cholesterol, HDL-cholesterol and triglycerides
- urine biochemistry (urine albumin/creatinine ratio)

These samples will be analysed in the LCC's local NHS laboratory.

Two questionnaires, namely Quality of Life (EQ-5D) and visual function (VFQ-25), will be completed on paper. Participants will be given an appointment to return to the LCC in approximately 8 weeks' time and definitely within 6-11 weeks. Any patients found to be ineligible at the time of the screening visit will be informed of this by the LCC research nurse and the CCO will mail a letter to the relevant GP to provide this information.

4.5.2 Consent

The LCC's local principal investigator (PI) carries overall responsibility for ensuring informed consent is obtained before any protocol specific procedures are carried out; however, responsibility will be delegated to the LCC research nurse and it is anticipated that the LCC research nurse will obtain consent from most, if not all, screened patients. The decision of a participant to participate in clinical research is voluntary and should be based on a clear understanding of what is involved.

The trial will be described in detail to the potential participant and will cover all the elements specified in the abbreviated PIL (only for participants recruited via DES results mail-out), the detailed PIL and the Informed Consent Form (ICF). The participant will be given every opportunity to clarify any points they do not understand and, if necessary, ask for more information. The participant will be informed that their medical records may be inspected by Regulatory Authorities and the Sponsor. The potential participant will have had days to decide about taking part in the trial after receiving the detailed PIL and discussing the trial with the research nurse on the telephone.

The research nurse or doctor obtaining consent and the participant will sign and date the ICF to confirm that consent has been obtained. The participant will receive a copy of this document, the original copy of the ICF will be stored in the Investigator Site File and a copy of the completed ICF will be provided to the CCO. A scanned electronic copy will be uploaded to the health board's electronic health record in those health boards which are using electronic health records. In any

health boards not using electronic records, a paper copy will be filed in the hospital case notes. The participant will be provided with a Treatment Information Leaflet.

No biological (tissue) specimens are being retained in the trial. However, participants will be asked to give permission for their digital retinal images to be stored as part of an imaging biobank to allow subsequent research. In addition, participants will be asked for their permission to use electronic health records (both those described in the protocol and other currently unspecified NHS datasets) to follow them up during the active treatment phase of the trial (i.e. the main study described in this protocol) and after its completion (i.e. post-trial long term follow-up). Long term follow-up is not covered by this protocol and it will require the development of a further protocol along with further ethical and PBPP approval. However, given that some microvascular outcomes such as blindness and amputation may take decades to occur, provision of consent will allow us to address these outcomes at a later stage.

4.5.3 Activities after the screening visit including local PI sign-off of eligibility

Along with the signed ICF, the LCC research nurse will upload a copy of the detailed PIL to the health board's clinical portal (i.e. electronic health record) to enable clinicians involved in the patient's care to be aware of, and gain further information about, the trial. In health boards which use paper records, the detailed PIL will be filed in the patient's case notes.

When the results of blood biochemistry (renal function, liver function tests [LFT], CK), HbA1c, random lipid profile [including at least total cholesterol, HDL-cholesterol and triglycerides]) are available these will be transcribed into the TMS and a further assessment made of the patient's eligibility. Urine biochemistry results will be obtained subsequently as part of SCI Diabetes data linkage. Local laboratory ULNs will be used for LFTs and CK and these will be entered into the eCRF. Local lab ULNs are not required for renal function, lipids, HbA1c. Validation queries will highlight clear errors and data will be obtained from SCI Diabetes at regular intervals (every 6-12 months) to retrospectively verify the accuracy of the transcribed data.

Based on the details collected at the screening visit and following receipt of the screening biochemistry results, eligibility for participating in LENS will be determined according to the eligibility criteria. This will be recorded electronically on the eCRF. Both patients and their GPs will be written to if their screening blood test results indicate that they are not eligible to enter the trial to explain this. For those patients who remain eligible, the local PI or a doctor to whom responsibility has been delegated will be required to sign off their eligibility. This sign-off will approve the participant to enter the run-in phase and to be randomised after the run-in if their biochemistry results at the Randomisation visit are within pre-specified limits (see below) (also termed an 'order-to-supply'). This approval will be in the form of an electronic signature on the trial eCRF. The local PI or delegated doctor's electronic sign-off will remain valid for the entire trial unless the clinical condition of the patient requires a reduction in dose or cessation of the study drug after randomisation. The 'order-to-supply' will be an inbuilt part of the eCRF.

Eligible patients will receive a letter/email from the CCO informing them that they have been entered into the run-in period, that a supply of open-label fenofibrate will be mailed to them shortly, informing them how often to take the study medication and requesting that all unused run-in study medication be returned to the LCC at the randomisation assessment. A letter will also be sent to the patient's GP by the CCO informing them of their patient's interest and likely participation in the trial, and providing the GP with an opportunity to exclude their patient if they feel that the patient's participation is inappropriate and a deadline for notification to the CCO. An automated text or email (depending on participant preference) will be sent to the participant informing them that study

medication is about to be sent to them and informing them of action to take if the study medication does not arrive shortly. Medication will then be mailed to the patient by routine post.

The LCC research nurse will contact the participant approximately 1-2 weeks after run-in study medication has been mailed to verify that it has been safely received, to obtain and record the study medication verification code from the participant (which will be entered into the eCRF) and to verify appointment details for the planned randomisation assessment.

4.5.4 Screen failures

Individuals who are screen failures (i.e. who attend screening visits and are found to be ineligible) will not be allowed to be re-screened.

Participants will receive reasonable travel expenses, including return taxi journeys where needed, but no additional funds.

4.5.5 Active run-in treatment

Participants who have been confirmed as eligible will be sent a 10-week supply of open-label fenofibrate tablets to be taken as one 145mg tablet daily if screening eGFR ≥60mL/min/1.73m^2 or one 145mg tablet every 2nd day if baseline eGFR 40-59mL/min/1.73m^2. The drug will be sent by routine post with instructions on how to take the tablets. GPs of any patients who drop out during the run-in phase or who ultimately fail to attend a randomisation visit will be informed of this by letter. It is thought highly unlikely that an active run-in with fenofibrate will impact upon retinal imaging in any material and sustained way.

4.5.6 Randomisation visit

Participants who enter the run-in will be seen approximately eight weeks (limits 6-11 weeks) after the initial screening visit. At this visit, all concomitant medications, compliance with the run-in treatment, and adverse events (including all serious adverse events; and any non-serious adverse events leading to the discontinuation of study treatment), will be recorded. Height, weight and vital signs (pulse rate and blood pressure) [average of 2 measurements for BP] will be measured and recorded. A blood sample for blood biochemistry will be taken and sent to the local laboratory to measure renal function, LFTs (including at least one of ALT or AST) and CK. Patients will be asked about any treatment-related side-effects that would prevent them from wanting to take part; to verify that they remain willing to take part in the trial; and that they have taken approximately 75% of the study medication or more to date. The LCC research nurse will have discretion to evaluate compliance if there are reasonable extenuating circumstances. Patients will be asked to stop taking the open-label fenofibrate. The LCC will dispose of any unused run-in fenofibrate which is returned. For any patients who do not wish to proceed to randomisation at this stage, their GP will be informed by letter from the CCO. Randomisation will only be possible when results of the blood tests from the visit become available, so will not occur immediately. Eligible patients will be given a Patient Participation Card.

It is possible that some participants having been recruited into the trial via HIC mail-out or the SDRN research register will receive a further invitation to participate via mail-out with their DES results. To avoid confusion, it will be explained at the Randomisation visit that these abbreviated PILs should be ignored.

4.5.7 Random allocation to fenofibrate or placebo

LENS will be a double-blind trial. Therefore, trial participants, investigators, and trial staff will be blinded to intervention group. Matching fenofibrate and placebo tablets will be used. Patients who remain eligible based on their Randomisation visit blood results and on-going consent will then be

randomised by the research nurse or local PI at the LCC via the CCO's TMS. Eligible participants will be randomised 1:1 to double-blind:

- Fenofibrate 145mg, or
- matching placebo

After the visit, participants will be mailed their first pack of medication containing a 26 week supply of study drug along with relevant information about the medication and the CTSU Freephone number for any trial-related queries from them or professionals responsible for their care. Participants with eGFR ≥60mL/min/1.73m^2 at the Randomisation visit will be asked to take one tablet daily, while participants with eGFR 30-59mL/min/1.73m^2 at the Randomisation visit will be informed to take one tablet of study drug every second day. Participants will be asked to take the study drug in the morning but they will be allowed to take the study drug at another time of the day if they prefer.

4.5.8 The randomisation scheme

A minimisation schedule of randomisation will be employed. Minimisation criteria will include: age, type of DM (type 1, type 2, other), sex, baseline DES grading, HbA1c, renal function and statin use. The randomisation algorithm will also contain a stochastic element.

4.5.9 Method of implementing the allocation sequence

A web-based randomisation system will be used for the trial and the server will be based at the CCO in the UK. The TMS will provide an immediate allocation to the relevant research nurse or doctor.

4.5.10 After the Randomisation visit

Patients whose Randomisation visit eGFR is <30mL/min/1.73m^2, CK is >3X ULN and/or ALT or AST >2.5X ULN will not be eligible to continue. Results will be transcribed into the eCRF when available to the LCC (preferably within one week), thereby allowing eligibility to be definitively determined according to the TMS. For patients who are now ineligible based on blood results, a letter of appreciation will be mailed to them from the CCO and their GP will also be informed by letter from the CCO. Likewise, those who remain eligible will be written to by the CCO to confirm this. A letter will also be sent from the CCO to the participant's GP advising that the individual is participating in LENS, providing details of the trial and also providing results for the randomisation visit blood tests.

4.6 POST RANDOMISATION FOLLOW-UP

4.6.1 Mailed medication and questionnaires

Participants will be mailed regular 26 week supplies of study drug starting immediately after randomisation (see Section 4.11.4). As with the run-in study medication, texts or emails (according to the preference of each participant) will be sent at the time of mailing out study medication informing participants that it should be received shortly and providing information on what action to take if not received. Participants will also have access to the CCO's 24 hour Freephone number and will be able to contact the CCO at any time with any concerns. Trial-specific questionnaires will be administered by telephone (with the option of follow up based on review of medical records where a participant cannot be contacted) while EQ-5D and VFQ-25 questionnaires will be administered on paper at set intervals as detailed in section 4.7. The LENS trial website

(https://www.ctsu.ox.ac.uk/lens) will provide information regarding the progress of the trial and newsletters will be sent to participants updating them about the progress of the trial.

4.6.2 Unplanned follow-up visits

No face-to-face visits are planned after randomisation. However, participants will be able to attend the LCC for a blood test to confirm their renal function status if there is any clinical concern over their continuation on allocated study drug.

4.6.3 Modifying study treatment

It is well established that fenofibrate leads to an increase in serum creatinine, typically by 10-20%[26]. Lower doses of fenofibrate are recommended in those with chronic kidney disease[27]. It is therefore expected that some trial participants initially taking one tablet daily will need to reduce their dose of study drug based on renal function in routinely collected blood tests.

During follow-up, renal function (based on routinely collected blood tests done at the discretion of each participant's GP and/or hospital doctor) will be monitored for safety in almost 'real time' using a bespoke SCI Diabetes domain created by SCI Diabetes. This will enable an experienced study doctor at the RCC to identify any LENS participants who cross pre-specified eGFR thresholds (namely 30 and 60mL/min/1.73m^2 respectively) within days of this occurring. To achieve this, the study doctor will perform an automated search of SCI Diabetes for any renal function blood results for all LENS participants approximately every 2 weeks. Participants whose Randomisation visit eGFR was ≥60mL/min/1.73m^2 (and who are therefore initially prescribed one study tablet daily) but whose on-treatment eGFR subsequently falls to 30-59mL/min/1.73m^2 in routinely collected blood tests will be asked to reduce study drug to one tablet every second day and no increase in dose frequency (i.e. back to one tablet daily) will be permitted thereafter. The LCC's local PI or LCC doctor with delegated responsibility or experienced CCO doctor will be required to electronically sign an updated 'order to supply' to record any such change. Participants whose on-treatment eGFR falls below 30mL/min/1.73m^2 will be asked to discontinue study drug therapy. Participants who experience a transient worsening in renal function due to a self-limiting illness (e.g. gastro-enteritis) during which study treatment has been stopped will be able to restart study medication subsequently in accordance with their renal function.

LFTs cannot be monitored in 'real time' in the same way via SCI Diabetes though ALT will be sought via regular SCI Diabetes data linkage. However, based on ACCORD-Lipid data (provided by Dr Henry Ginsberg and Dr Laura Lovato) we would only expect to have one additional participant on fenofibrate vs. placebo with ALT >5X ULN over 5 years' post-randomisation follow-up in LENS. This also assumes that fenofibrate treatment can cause deteriorations in LFTs in some patients, an assumption which remains unproven.

Temporary breaks from taking study treatment, whereby a participant indicates that they wish to stop taking study tablets for a period of time, are allowed. Changes of consent in the trial, including withdrawal, are discussed in section 4.8.

4.6.4 Follow-up of outcomes by electronic linkage, questionnaire and health records

Data will be sought by electronic linkage, by questionnaire and by checking health records as described in Table 3. Linkage data will either be obtained from Scotland's electronic Data Research and Innovation Service (eDRIS) at agreed intervals or from the relevant registries (in accordance with Research Ethics Committee [REC] and Public Benefit and PBPP approvals). Data linkage may be considered via other NHS registries for any participants who relocate elsewhere in the UK.

Table 3. Sources of pre-specified outcome and other data

**Primary and Secondary Outcomes**	
Progression of DR or DR-related interventions	DES, SCI Diabetes system (these datasets are updated at least weekly); data will be sought every 6-12 months
DR-related interventions	SCI Diabetes system; trial-specific questionnaire (with uploading of supporting data from local NHS health records to TMS by LCC to allow adjudication); data will be sought every 6-12 months from SCI Diabetes and trial-specific questionnaires will be sent every 6 months
Visual acuity	DES, SCI Diabetes system; data will be sought every 6-12 months
Hard exudates within 1 disc diameter (M2)	DES, SCI Diabetes system; data will be sought every 6-12 months
Macular oedema	OCT analyses
Questionnaires	EQ5D, VFQ-25 and trial-specific questionnaires (administered by telephone); EQ-5D and VFQ-25 to be administered after 2 years and trial end; and trial-specific questionnaire every 6 months and trial end
Total cost to the health Service	Data on health service resource use from study case report forms, patient questionnaires, routine datasets (SCI Diabetes system, Scottish Morbidity Record (SMR) 01, SMR00) combined with national unit cost data.
Incremental cost-effectiveness	Based on total costs to the health service and QALYs estimated from participant responses to the EQ-5D
**Tertiary Outcomes**	
Cardiovascular events	SMR01, trial-specific questionnaire*, report to Freephone service (*for verification by LCC using manual search of NHS health records); data linkage to SMR01 to be conducted 2-3X during the trial, trial-specific questionnaires to be sent every 6 months
Lower limb non-traumatic amputation	Office of Population Censuses and Surveys (OPCS) codes within SMR01, trial-specific questionnaire*, report to Freephone service (*for verification by LCC using manual search of NHS health records); data linkage to SMR01 to be conducted 3X during the trial, trial-specific questionnaires to be sent every 6 months
Urine albumin creatinine ratio	SCI Diabetes system; data will be sought every 6-12 months
**Safety, other events of interest, and prescribing**	
Mortality	National Records of Scotland (NRS) Death Records, reports to CCO; patients will be flagged with NRS to ensure prompt recognition of any death

Hospitalisations	SMR01, questionnaire, report to Freephone service; data linkage to SMR01 to be conducted 2-3X during the trial, trial-specific questionnaires to be sent every 6 months
Biochemistry	SCI Diabetes system; data will be sought every 6-12 months
Prescription data	Prescribing Information System (PIS); data will be sought 2-3X during the trial

4.6.5 Long term follow-up assessments

Consent will be sought from participants to allow extended follow-up after the main study (described in this protocol) is concluded, thereby providing valuable information on the longer-term safety and efficacy of the study treatment. This can be particularly informative for assessing effects on outcomes that may only become evident many years after initiation of treatment, and for deriving appropriate cost-effectiveness analyses. However, a further protocol will be developed and further ethical approval and PBPP approval will be required before such work is undertaken. Any long term follow-up is likely to be achieved using the same electronic registry sources detailed in this protocol and potentially other NHS registry sources also. Outcomes of interest are likely to include (but may not be limited to) the current trial primary, secondary and exploratory outcomes.

4.6.6 Unblinding

Unblinding of treatment allocation (fenofibrate or placebo) for an individual participant is warranted when knowledge of the treatment allocation could materially influence the immediate medical management. Urgent unblinding is available via a 24-hour Freephone service at CTSU. Requests for unblinding will be reviewed urgently and authorised by the CCO on-call clinician. For the avoidance of doubt, where an investigator or treating physician requires that a participant under their care is unblinded, the CCO will unblind the participant and provide this information.

4.6.7 Post-trial care

When study treatment stops at the end of the trial, it will no longer be available to participants unless it is prescribed by their usual doctor.

4.7 TRIAL QUESTIONNAIRES

During LENS, participants will be asked to complete regular questionnaires including the EQ-5D, VFQ-25 and a trial-specific follow-up questionnaire. The EQ-5D and VFQ-25 will first be completed by the participant during the Screening visit on paper; and then after 2 years and at the final assessment. The trial-specific questionnaire will first be completed by telephone 6 months after the randomisation visit and then every 6 months by telephone thereafter and at the end of the study as described below. Follow up by medical records will be available when participants cannot be contacted. EQ-5D and VFQ-25 will be completed by participants on paper. The RCC and CCO will support the LCC with the conduct of telephone interviews for questionnaires where necessary.

4.7.1 EQ-5D

This is a standardised and validated instrument for use as a measure of health outcome in which the respondent is asked to indicate his/her health state by selecting the most appropriate statement in each of five dimensions[28].

4.7.2 VFQ-25

This is a standardised and validated questionnaire which measures the dimensions of self-reported vision-targeted health status that are most important for persons who have chronic eye diseases[29].

4.7.3 Trial-specific follow-up questionnaire

This questionnaire will require participants to verify their contact details and GP details; to provide information regarding their compliance with study treatment and their willingness to continue taking the study treatment; to provide information about any contra-indicated medications; and to report on specific health events of interest (eye procedures related to DR; lower limb amputation; cardiovascular events); and any other serious illnesses or hospital admissions. In addition, information will be collected regarding receipt of trial medication by post and study medication pack information (pack numbers, verification codes) for received study treatment packs will be recorded wherever possible.

4.8 STOPPING STUDY TREATMENT, CHANGE OF CONSENT AND WITHDRAWAL

4.8.1 Stopping Study Treatment and Change of Consent

Study drug will be discontinued if a clear contra-indication to its continued use is reported by a participant, by their doctor, or if such information becomes available from linkage to electronic health records. Circumstances under which discontinuation of study treatment will occur include (but may not be limited to) the following:

- a Suspected Serious Adverse Reaction (SSAR) (as defined in section 5.1) or Suspected Unexpected Serious Adverse Reaction (SUSAR) (as defined in section 5.1) is reported
- eGFR <30mL/min/1.73m^2 (Section 4.6.3)
- Reporting of the following adverse events (pancreatitis, gallbladder disease [cholecystitis, cholecystectomy], severe liver disease, rhabdomyolysis or myopathy, commencement of renal replacement therapy)
- Commencement of a contraindicated medication (Section 4.11.10)
- Overdose of study treatment (Section 5.4.3)
- Pregnancy (Section 5.4.2)
- Discontinuation of adequate contraception by a female participant of child-bearing potential (Section 4.11.12)

In addition, patients may stop their allocated study treatment at any time and for any duration without necessarily giving a reason but reasons for discontinuation will be sought and recorded directly in the database; in such cases, these participants will be able to recommence study treatment any time at their own request if there is no contra-indication to fenofibrate therapy. Unless a participant specifically withdraws permission for all forms of follow-up, he/she will continue to be followed up via their electronic records irrespective of whether he/she continues to complete questionnaires.

4.8.2 Withdrawal

A participant can withdraw from the trial at any time. Withdrawal indicates that the participant no longer wishes to take the study drug and that he/she does not agree to ongoing follow up of any kind (including telephone follow-up, emailed questionnaire and electronic linkage). There is no obligation for a participant to provide signed confirmation of his/her withdrawal. Information collected about the participant up to the date of withdrawal will still be retained and analysed but no further data will be sought or stored from that date forwards.

4.9 ANALYSIS OF BLOOD AND URINE SAMPLES

No biological samples will be retained or stored during the trial for research purposes. All blood and urine samples for assessing eligibility and for research purposes taken at the screening visit and randomisation visit will be sent to the LCC's local NHS hospital biochemistry laboratory for analysis. These samples will be discarded or destroyed by these laboratories according to their standard operating procedures. LCCs will be responsible for deciding how many blood samples need to be sent to their local laboratory for the required analyses in accordance with their laboratory policy. Typically, this will be two whole blood samples (one gel tube for renal function, LFTs, CK, and lipid profile [hours since last meal will be recorded; there is no requirement to fast]; one EDTA tube for HbA1c analysis) and one spot urine sample (in plain tube) at the screening visit; and this will be followed by one blood sample (one gel tube for renal function, LFTs, CK) at the randomisation visit which again does not require fasting. All relevant NHS laboratories have Clinical Pathology Accreditation according to International Standard for Organisation 15189 and currently perform regular quality control procedures for the routine assays in question. Biochemistry results taken at the screening and randomisation visits will be transferred to the TMS via two routes: either by LCC staff manually entering the data directly into the eCRF or by linkage to the SCI Diabetes registry and uploading the data.

Unplanned visits to local sites by participants after randomisation can be arranged by the CCO, RCC or LCC for the purposes of biochemistry safety testing if this is felt necessary to determine the safety of ongoing treatment with the study drug.

4.10 END OF TRIAL

The trial will continue until at least 222 primary events have occurred and until median follow-up is at least 4 years, whichever is the later. The 'end of study' is defined as the date on which the trial database is locked for the primary analysis. The Trial Steering Committee (TSC) has the right to terminate the study for clinical or administrative reasons at any time.

The end of the study will be reported to the REC and Regulatory Authority within 90 days, or 15 days if the study is terminated prematurely. Investigators will notify participants and inform them of appropriate procedures to follow. A summary report of the study will be provided to the REC and Regulatory Authority within one year of the end of the study.

4.11 TRIAL MEDICATION

4.11.1 Name and description of investigational medicinal product(s) (IMP)

IMP name:	Lipantil Supra 145 mg film coated tablet [fenofibrate (nanoparticles) 145 mg].
Marketing Authorisation Holder:	Mylan IRE Healthcare Limited, Unit 35/36, Grange Parade, Baldoyle Industrial Estate, Dublin 13, Ireland
Marketing Authorisation Number:	PA 2010/015/003

The IMP to be used in LENS is fenofibrate in the form of 145mg film-coated tablets with a nanoparticle formulation. The control will be a matching placebo tablet. The marketing authorisation for this formulation of fenofibrate is held by Mylan in the Republic of Ireland and other member states of the European Economic Area (EEA) though not in the UK. Study IMP and matching placebo tablets will be supplied in bulk form by Mylan free of charge.

4.11.2 Legal status of the drug

Fenofibrate is a licensed drug in the UK to treat “mixed hyperlipidaemia if statin contra-indicated or not tolerated, or in severe hypertriglyceridaemia; or as an adjunct to statin in mixed hyperlipidaemia if triglycerides and HDL-cholesterol inadequately controlled in patients at high cardiovascular risk”[27]. In LENS the drug will be used outside these indications, and we will use a formulation currently in use elsewhere in the EU which is bio-equivalent to doses used in the UK. Notably, Australia and Singapore have licensed fenofibrate for participants with DR, the same group to be studied in LENS.

4.11.3 Summary of Product Characteristics (SmPC)

The relevant section of the SmPC for fenofibrate will be used as the Reference Safety Information during the trial for pharmacovigilance reporting purposes. The sponsor will periodically check (at least once a year) for any changes to the licensing status of fenofibrate.

4.11.4 IMP preparation, storage, labelling and supply

IMP will not be issued to study participants at face-to-face study clinic visits, instead all study treatment will be issued from a central location and sent to study participants, on instruction from the CCO, via a mailing service.

Two different types of packaged study IMP will be used (see Table 4 below).

- **Run-in Treatment** will be issued and sent to consenting participants after the initial Screening visit (once confirmed eligible by their blood results) to be taken during the pre-randomisation Run-in phase of the trial.

- **Randomised Treatment** will be sent to participants following their Randomisation visit, new supplies sent at approximately week 22 post randomisation and at 26-weekly intervals thereafter. Participants will be sent a Randomised Treatment pack (Type A or Type B; see Table 4) corresponding to their most recently recorded renal function.

Table 4. Run-in treatment and randomised treatment to be used in LENS

Treatment Dose	Run-in treatment	Randomised treatment
fenofibrate 145mg (open label)	**One pack** containing 70 tablets	n/a
fenofibrate 145mg **OR** placebo (double blind)	n/a	**Type A pack** containing 180 tablets (for participants with most recent recorded eGFR of ≥60/mL/1.73m^2
fenofibrate 145mg **OR** placebo (double blind)	n/a	**Type B pack** containing 90 tablets (for participants with most recent recorded eGFR 30-59mL/min/1.73m^2)

Mylan will provide tablets of fenofibrate and matching placebo in bulk form. Batches of tablets will be delivered according to an agreed schedule and in agreed quantities to the trial's contract packager (details below):

Contract packaging company:	AndersonBrecon (UK) Limited
Address:	Pharos House, Wye Valley Business Park, Brecon Park, Brecon Road, Hay-on-Wye, Hereford, HR3 5PG, UK
Authorisation number:	MIA(IMP) 11724

Under the direction of the CCO the contract packager will be responsible for:

- packaging, labelling and QP release of uniquely identifiable packs of finished study IMP in accordance with EU Good Manufacturing Practice (GMP) standards
- storage of released packaged IMP
- selection and preparation for despatch of specified bottles of packaged IMP assigned to specified study participants

Royal Mail (along with other third party mailing/courier service provider(s) if required) will be responsible for delivering packs containing specified bottles of packaged IMP assigned to specified study participants. All packs of study IMP sent to study participants will be sent by routine post. At the time of mailing out IMP (including both run-in packs and post randomisation packs), participants will receive an automated text or email informing them that their study treatment is *en route* and to contact the CCO if the study treatment does not arrive safely.

Design and formatting of IMP labels required for the two types of Treatment Bottle will be the primary responsibility of the contract packager in collaboration with the CCO. All IMP labels will be designed in accordance with Annex 13 of the EU GMP Guide.

The CCO will maintain an inventory and audit trail of all bottles of study IMP on the TMS that have been requested, packaged, assigned and sent to participants, returned by participants and destroyed. In addition, the TMS will record any bottles of study IMP that have expired or been damaged prior to being assigned to study participants as well as a record of their subsequent destruction.

Participants will be advised to store study IMP at room temperature (Store below 30°C).

All bottled study IMP will be labelled with an expiry date beyond which it should not be used, and will only be issued to participants with due allowance for the remaining shelf life.

Participants will be asked to return unused Run-in drug to the LCC at the time of the Randomisation visit and the LCC will be responsible for its handling and destruction. When required, study participants will be advised to take any expired or unused study IMP to a local pharmacy for safe disposal. This goes against normal practice for return and accountability of IMP in standard clinical trial settings. However, due to the streamlined design of the LENS trial, where participants will not attend routine clinics during the follow-up phase of the trial, it has been deemed to be too problematic and prone to unacceptable risk to expect study participants to securely return study IMP via the postal system or other courier service.

The CCO will be responsible for handling and destruction of any returned, expired or unused IMP and packaging materials which it may receive back from study participants or that is returned as undelivered. Likewise, LCCs will be responsible for handling and destruction of any returned, expired or unused IMP and packaging materials which are handed in to them.

4.11.5 Dosage schedules

The dose of fenofibrate tablets is 145mg in nanoparticle formulation. After randomisation, study drug will be taken orally as one tablet daily (in the context of normal renal function i.e. eGFR ≥60mL/min/1.73m^2) or one tablet every second day (in the context of chronic kidney disease with eGFR 30-59mL/min/1.73m^2). Study drug can be taken with or without food[30].

Doses of fenofibrate used in earlier trials were 200mg daily (micronised) in FIELD and 160mg daily in ACCORD-Lipid. The selected formulation and dose for LENS is bio-equivalent to the doses used in FIELD and ACCORD-Lipid. Intermittent dosing is frequently used in clinical practice for other clinical conditions (e.g. vitamin D in chronic kidney disease). Give the lack of availability of lower dose fenofibrate tablets for LENS and, in the context of data suggesting that fenofibrate treatment may in fact be beneficial for patients with chronic kidney disease[31], intermittent dosing for those with chronic kidney disease represents a suitable option. No further study drug will be provided after the completion of the trial.

4.11.6 Fenofibrate and renal function

It is well established that fenofibrate therapy increases creatinine, that this effect is exerted quickly and that it is fully reversible[26,32.] British National Formulary (BNF) advice is to use a lower dose at eGFR 20-59mL/min/1.73m^2 (namely 134mg/d), a further reduced dose at eGFR 15-19mL/min/1.73m^2 (67mg/d) and to stop treatment below eGFR 15mL/min/1.73m^2 [27]. Notably, there is no evidence that those with the greatest increase in creatinine on fenofibrate experience any clinical disadvantage, the reduction in urine albumin on the drug suggests the possibility of renal benefit, and fenofibrate treatment actually slows the rate of the fall in eGFR over time[33,34]. In addition, there is evidence of potential cardiovascular benefit in patients with chronic kidney disease who are treated with fenofibrate[31]. Renal function will be checked at screening and after approximately eight weeks' open-label run-in treatment, providing information on safety. Thereafter, monitoring of renal function in LENS will be based on results from blood samples taken as part of routine clinical practice. Scottish Diabetes Survey data indicate that 94% with DM have renal function checked every 15 months[35.] Both the eGFR thresholds and doses used in the trial are also more conservative than what is recommended in the BNF. In addition, participants can attend the LCC for a blood test to confirm their renal function status if there is any clinical concern over their continuation on allocated study drug. All biochemistry laboratories in NHS Scotland report both serum creatinine and eGFR.

4.11.7 Dosage modifications

Participants whose Randomisation visit eGFR (i.e. after open-label treatment with fenofibrate) is ≥60mL/min/1.73m^2 and whose eGFR falls to 30-59mL/min/1.73m^2 thereafter while on randomised study treatment will be required to reduce study drug to one tablet every second day (see section 4.6.3). The local PI or clinician with delegated responsibility or experienced CCO clinician will be required to electronically sign an updated ‘order to supply’ to verify this. Participants whose eGFR falls below 30mL/min/1.73m^2 will be asked to discontinue study drug therapy (see section 4.6.3). Dosage will not be reduced based on patient request alone. Participants who experience a transient worsening in renal function due to a self-limiting illness (e.g. gastro-enteritis) during which study treatment has been stopped will be able to restart study medication in accordance with their renal function.

4.11.8 Plan for study treatment after the occurrence of a pre-specified outcome

Participants should keep taking allocated study treatment if a primary, secondary or tertiary outcome occurs unless any contra-indication has developed or if a doctor responsible for their care wishes to prescribe a fibrate.

4.11.9 Contra-indications

Exclusion criteria of the trial are in accordance with the SmPC which lists the following conditions as contra-indications − hepatic insufficiency (including biliary cirrhosis), renal insufficiency, children, hypersensitivity to fenofibrate or any component of this medication, known photo-allergy or phototoxic reaction during treatment with fibrates or ketoprofen, gallbladder disease, chronic or acute pancreatitis; no suitable data regarding use in pregnancy and/or lactation exist.

4.11.10 Concomitant medications and potential interaction with other therapies

If a participant commences any fibrate, vitamin K antagonist therapy (warfarin, acenocoumarol or phenindione), cyclosporin (an immunosuppressant), colchicine (gout medication), ketoprofen (NSAID), daptomycin (antibiotic) or rosuvastatin (only 40mg daily) therapy during LENS, they will need to discontinue fenofibrate but they will remain under active follow-up. Participants will be asked about such therapies during post-baseline questionnaires. If such a treatment is subsequently discontinued at any later point in the trial, the participant will be asked by the research team for their permission to recommence study treatment. A post hoc analysis of the ACCORD-Lipid trial found that a greater number of participants allocated to fenofibrate developed a low HDL-cholesterol level compared to placebo recipients (5% absolute excess) and that this was limited to participants receiving a glitazone[36]. It has not been demonstrated that this biochemical finding increases clinical risk. It is anticipated that only approximately 8% (n=80) of LENS participants are likely to be treated with a glitazone indicating that this effect, if true, should only occur in fewer than five participants on fenofibrate. The numbers of participants who have developed a HDL-cholesterol level ≤0.60mmol/L will be reported to the DMC at approximately one year after the completion of randomisation.

4.11.11 Concomitant statin therapy

Many adult patients with DM in Scotland are already on statin therapy. Combination therapy consisting of gemfibrozil and statins is contraindicated based on an increased risk of rhabdomyolysis[37,38]. This led to previous concerns about a fenofibrate-statin combination however there is nowadays widespread clinical use of the combination of statins and fenofibrate. The ACCORD-Lipid trial, a double-blinded RCT comparing fenofibrate plus simvastatin to simvastatin plus placebo in 5,518 patients with type 2 DM, found no increased rates of any serious side-effects including muscle-related side-effects, whether defined in terms of reports of muscle pain, recorded

SAEs (myopathy or myositis) or increases in CK levels to >5X or >10X normal among those on statin plus fibrate versus statin alone[13]. In ACCORD-Lipid, the number of participants developing elevated ALT was: ALT >3X ULN 52 (1.9%) vs. 40 (1.5%) cases (non-significant) and for ALT >5X ULN 16 (0.6%) vs. 6 (0.2%) cases (borderline significant; ALT >3X ULN includes these 22 cases with ALT >5X ULN). Systematic reviews of the effects of fenofibrate on a variety of possible side-effects show no increased rate of side-effects on the combination of statin and fenofibrate[39]. Fenofibrate also does not affect the pharmacokinetics of simvastatin or atorvastatin[37,40]. The safety of combination therapy with a statin is underlined by the UK's licensing which allows the use of fenofibrate as an adjunct to statin therapy in certain groups of patients, as well as by the availability of preparations containing both fenofibrate and simvastatin. However, based on regulatory feedback, patients taking rosuvastatin 40mg daily will be excluded from the trial and any participant who commences rosuvastatin 40mg daily during follow up will be required to discontinue study treatment.

4.11.12 Trial restrictions

There are no dietary or lifestyle restrictions during the trial.

Female participants of child-bearing potential must be using adequate contraception for the duration of the trial and for at least 7 days after discontinuing the study drug, whether due to the end of the trial or withdrawal. Adequate contraception includes:

- Intrauterine Device
- Hormonal based contraception (pill, contraceptive injection etc.)
- Double Barrier contraception (condom and occlusive cap e.g. diaphragm or cervical cap with spermicide)
- True abstinence: when this is in line with the preferred and usual lifestyle of the subject

4.11.13 Assessment of compliance

Compliance with open-label active run-in fenofibrate will be assessed at the randomisation visit. Thereafter, randomised participants will be asked about their compliance to treatment in six monthly trial-specific questionnaires.

4.11.14 Drug mail-out

As noted above, LENS study medication will be sent to participants by routine post, namely by Royal Mail which is the only provider obliged to provide coverage to the whole of mainland Scotland. Other ongoing or recently completed randomised trials in cardiovascular disease in the UK such as ASCEND (https://ascend.medsci.ox.ac.uk; n=15,000 randomised to aspirin or placebo; run by CTSU)[21], FAST (http://www.fast-study.co.uk; n=5,700 to assess the cardiovascular safety of feboxustat) and GLINT (https://www.dtu.ox.ac.uk/GLINT/; n=250 randomised to metformin or placebo; jointly run by the Diabetes Trials Unit, University of Oxford) have also sent study medication by routine post. Various steps will be taken to ensure the reliability of this method of delivery and these are described in the relevant parts of the protocol. These steps include: (i) phone calls by LCC nurses during run-in to verify receipt of study medication and to collect and record the study medication verification code (ii) automated texts/emails in parallel with study medication mail-out to inform participants that their study medication is en route and requesting that they contact the CCO if it is not received (iii) asking about receipt of study medication plus, where possible, study medication verification codes as part of six monthly trial-specific follow-up questionnaires. In addition, LENS participants will have access to an experienced CCO clinician via the CTSU Freephone number at any time.

5 PHARMACOVIGILANCE

5.1 DEFINITIONS TO BE USED IN LENS

Table 5. AE definitions to be used in LENS

Term	Definition
Adverse Event (AE)	Any untoward medical occurrence in a participant to whom a medicinal product has been administered, including occurrences which are not necessarily caused by or related to that product.
Adverse Reaction (AR)	An untoward and unintended response in a participant to an investigational medicinal product (IMP) which is related to any dose administered to that participant
Serious Adverse Event (SAE)	A SAE is any untoward medical occurrence that:<br>• results in death<br>• is life-threatening<br>• requires unplanned inpatient hospitalisation (excluding A&E attendances without admission to a hospital ward) or prolongation of existing hospitalisation<br>• results in persistent or significant disability/incapacity<br>• consists of a congenital anomaly or birth defect<br><br>Other ‘important medical events’ may also be considered serious if they jeopardise the participant or require an intervention to prevent one of the above consequences.
Suspected Serious Adverse Reaction (SSAR)	An AE that is both serious and, in the opinion of the reporting Investigator or clinician, believed with reasonable probability to be caused by the trial treatment, based on the information provided.
Suspected Unexpected Serious Adverse Reaction (SUSAR)	A serious AR, the nature and severity of which is not consistent with the information about the medicinal product in question set out in the SmPC.
Overdose	Any occasion in which a member of the research team learns that a participant has taken at least 6 study tablets within a 24 hour period

5.2 EVENTS ASSOCIATED WITH FENOFIBRATE USE

The most commonly reported AEs during fenofibrate therapy are digestive, gastric or intestinal disorders. The SmPC provides a list of the undesirable effects that have been observed during placebo-controlled clinical trials and post-marketing studies. The SmPC will be reviewed at least annually for changes.

5.3 RECORDING AND REPORTING ADVERSE EVENTS

Fenofibrate is a well profiled medicine which has been studied intensively in major cardiovascular trials of over 15,000 participants and it is also licensed to treat patients with dyslipidaemia. In 2016, NHS Scotland data show that 18,600 NHS prescriptions were issued for fenofibrate (and 27,000 prescriptions for the closely related bezafibrate).

5.3.1 NSAEs and SAEs

Participants in LENS are not considered to be at higher risk of ARs than these groups. Information regarding the occurrence of non-serious AEs in this streamlined mail-based trial will therefore not be systematically collected unless they are medical eye procedures or they lead to the cessation of study treatment. Other reasons for cessation of study treatment will also be collected. Information on all SAEs will be collected and reported. The information that will be collected for AEs is:

- description of event
- seriousness
- date of onset
- end date (if resolved)
- an assessment of the relationship between study treatment and the AE (i.e. relatedness)

With the exception of AEs identified by registry linkage, where no assessment of relatedness is possible, relatedness will be determined by the LCC doctor and/or CCO doctor.

Information regarding AEs will be collected from the following sources:

- six monthly trial-specific questionnaires (by telephone or, where necessary, medical records)
- electronic linkage to health registry data and death records
- 24hr Freephone service
- manual searches of electronic health records
- LCC staff

Participants will have a card with details of the trial, CCO contact information and the Freephone number to facilitate reporting of any events. Data for AEs will be collected from the point of screening an individual up until the final registry linkages and questionnaires are completed.

SAEs brought to the attention of the LCC, whether during phone questionnaires or by ad hoc reports, will be reported to the Sponsor immediately (within 24 hours) of trial staff becoming aware of them. Such SAEs, and any SAEs reported via Freephone to the CCO, will be recorded in the trial's web-based CRF immediately. Reporting of SSARs is discussed in the next section. Verification of all SAEs will not be sought from health records with the following important exceptions:

- possible cardiovascular events
- possible lower limb amputations
- SSARs (including SUSARs)

Information regarding SAEs provided in trial-specific questionnaires will be assumed to be correct unless refuted by information from the participant's health records or health registry linkage. The CCO may request further information where information regarding a SAE is considered incomplete.

Procedures for verification and adjudication of potential study endpoints are described in section 3.3.

Experienced doctors within the CCO will review the blinded listing of all AEs on a regular basis (approximately daily). Tabulations of all SAEs and reasons for stopping study treatment will be provided to the DMC (unblinded) and the TSC (blinded) in the relevant reports. Information regarding AEs will be reported to Mylan in accordance with the legal agreement between the University of Oxford and Mylan.

5.3.2 SSARs

Any SAE that is considered, with a reasonable probability, to be due to study treatment is potentially a SSAR.[1] In making this assessment there should be consideration, based on:

- the available information,
- the probability of an alternative cause
- the timing of the reaction with respect to study treatment
- the response to withdrawal of the study treatment
- the response to subsequent re-challenge (where appropriate)

Any potential SSAR which comes to the attention of LCC staff must be reported to the CCO immediately (at least with 24 hours) of becoming aware of it. The following standard information will be obtained and recorded:

- Participant study number
- Identity of reporting person
- Event description
- Reason for attribution to study treatment

All such SSAR reports will be reviewed urgently by the Chief Investigator (or delegated deputy) at the CCO, who will assess the evidence for seriousness and relatedness, seek any additional information required (including relevant information relating to medical history and treatment both prior to and following randomisation), and assess expectedness based on the SmPC.

Any SSAR that is considered to be “unexpected” (i.e. a Suspected Unexpected Serious Adverse Reaction [SUSAR]) will lead to the relevant participant being unblinded. All SUSARs for participants randomized to active fenofibrate will be reported by the CCO to the MHRA and REC in an expedited fashion. For SUSARs that are fatal or life-threatening, an initial report will be submitted within 7 days (*7 day report*) of the date received, with a follow-up report provided within 8 days of the initial report, if required. For all other SUSARs, a 15 day report will be required. The following minimum information will be required for any SUSAR report:

- EudraCT number for the trial (2016-002656-24)
- Name of the suspected study medication (fenofibrate)
- Participant study number
- Identity of reporting person

---

[1] As recommended by regulatory authorities, a Suspected Serious Adverse reaction (SSAR) should be considered an unanticipated problem requiring expedited reporting only if it is either an event that is uncommon and strongly associated with drug exposure (such as angioedema, agranulocytosis, hepatic injury, or Stevens-Johnson syndrome), or an event that is not commonly associated with drug exposure, but uncommon in the study population (e.g. tendon rupture, progressive multifocal leukoencephalopathy).

- Event description
- Reason for attribution to study treatment
- Treatment assignment after unblinding

Confirmed reports of SSARs and SUSARs will be promptly forwarded to the chairman of the Data Monitoring Committee (DMC) and will be included in the Development Safety Update Report (DSUR) sent to the REC and MHRA. Study treatment will be discontinued in the event of a confirmed SSAR or SUSAR. Local PIs will be informed of any SUSARs which occur during LENS.

5.3.3 Reporting urgent safety measures

If the Protocol needs to be amended urgently because of a safety concern the CI/Sponsor will immediately, and in any event no later than 3 days from the date the measures are taken, give notice to the MHRA (initially by telephone followed by written notification) and the relevant REC of the measures taken and the circumstances giving rise to those measures.

5.3.4 The type and duration of the follow-up of subjects after AEs

Any participant experiencing a SSAR or SUSAR will be followed up for the remainder of the trial period and until the event has resolved (whichever is the longer).

5.3.5 Development Safety Update Reports

The CCO will submit DSURs once a year throughout the clinical trial or, on request, to the MHRA, the REC and the Sponsor.

5.4 NOTIFICATION OF SPECIFIC EVENTS

5.4.1 Deaths

If a death is reported by LCC staff or via the Freephone service, as much information as possible will be collected, a provisional cause of death assigned and it will be entered into the study database. SSARs or SUSARs will be reported as detailed in section 5.3.2.

5.4.2 Pregnancy

If a trial participant becomes pregnant, she will be required to discontinue study drug and information regarding her participation in LENS (including study treatment allocation) will be provided to her caring obstetrician. The outcome of the pregnancy will be followed up by the LCC. Information regarding pregnancy will not be collected for male participants whose partners fall pregnant during the trial.

5.4.3 Overdose

Any overdose of study medication (as defined in Table 5) should be reported to the CCO and sponsor according to the same timelines as SSAR notification. The participant will be required to discontinue study drug but will continue to be followed up in the trial.

5.5 RESPONSIBILITIES

5.5.1 LCC PI and LCC research nurse:

- Ensuring that details of SAEs and NSAEs that lead to the cessation of study treatment that are reported to the LCC are recorded in the TMS
- Ensuring that all SSARs (including any suspected SUSARs) that are reported to the LCC are recorded and reported to the CCO within 24 hours of becoming aware of the event and provide further follow-up information as soon as available.

5.5.2 Chief Investigator (CI):

- Clinical oversight of the safety of patients participating in the trial, including an ongoing review of the risk / benefit.
- Assessment of seriousness, relatedness and expectedness of potential SSARs and SUSARs.
- Immediate review of all SUSARs (this role will be fulfilled by a nominated deputy when the CI is unavailable).
- Review of SSARs in accordance with the trial risk assessment and protocol
- Assigning Medical Dictionary for Regulatory Activities (MedDRA) coding to all SAEs and SSARs.
- Preparing the clinical sections, reviewing the SmPC for any updates and final sign off of the DSUR.
- Unblinding of a participant for the purpose of expedited SUSAR reporting.

[Note: these responsibilities may be delegated to one or more suitably qualified deputies, but the CI always retains overall responsibility.]

5.5.3 CCO:

- Central data collection and verification of SAEs according to the trial protocol.
- Reporting safety information to the CI for the ongoing assessment of the trial's risk / benefit.
- Reporting safety information to the independent oversight committees identified for the trial (DMC and/or TSC) according to the Trial Monitoring Plan.
- Expedited reporting of SUSARs to the Competent Authority (MHRA in UK), and Sponsor and REC within required timelines.
- Notifying local PIs of any SUSARs that occur within the trial.
- Checking for and notifying local PIs of updates to the Reference Safety Information for the trial.
- Preparing standard tables and other relevant information for the DSUR in collaboration with the CI and ensuring timely submission to the MHRA and REC.

5.5.4 Trial Steering Committee:

The TSC will review safety data and liaise with the DMC regarding safety issues on a regular basis, with frequent meetings during recruitment, and with the option of unplanned meetings if required.

5.5.5 Data Monitoring Committee:

The DMC will annually review unblinded overall safety data to determine patterns and trends of events, or to identify safety issues, which would not be apparent on an individual case basis, with the option of more frequent meetings. There will also be a chairperson's review every 6 months.

6 STATISTICS AND DATA ANALYSIS

6.1 SAMPLE SIZE CALCULATION

Availability of prospective NHS Scotland DES data allowed progression rates for DR to be calculated directly from the target population in which LENS will be conducted. Data in Table 6 indicate DR progression rates (limited to patients with at least 3 years' follow-up and a baseline result prior to 2009). If it is assumed that participants will be recruited from among the various grades with observable DR in proportion to their prevalence, the progression rate from observable to 'referable' DR would be approximately 29% over 4 years. On that basis, 1060 participants would yield approximately 222 events over an average follow-up of 4 years, providing 85% power to detect a 33% reduction in progression (allowing for 15% drop-out) at alpha = 0.05. We aim to randomise at least 1060 participants.

As described in LENS' inclusion criteria, patients with R1 DR will need to have bilateral rather than unilateral R1 given that progression rates to referable DR are 2.4X and 3.7X higher in type 1 DM and type 2 DM respectively with bilateral R1 compared with unilateral R1. Inclusion of patients with unilateral R1 would substantially reduce event rates.

The LENS composite primary outcome consists of progression of observable DR to referable DR, or any of retinal laser therapy, vitrectomy or intra-vitreal injection of medication due to DR. While DR progression is likely to dominate in terms of numbers of events, this composite outcome has been selected to avoid missing any unexpected rapid progression of DR which requires intervention before the next DES screening visit.

Table 6. Progression rates of DR in Scotland (baseline data pre-2009)

	**Progression to referable retinopathy or maculopathy over 3 years***			
**Baseline grading**	**M2**	**R3**	**R4**	**M2 or R3 or R4**
**Type 1 DM (n=14,354)**				
R0 M0 (n=7,202)	131 (1.8%)	204 (2.8%)	12 (0.2%)	347 (4.8%)
R1 (one eye) M0 (n=2,480)	170 (6.9%)	21 (0.9%)	24 (1.0%)	215 (8.7%)
R1 (both eyes) M0** (n=4,016)	622 (15.5%)	121 (3.0%)	102 (2.5)%	845 (21.0%)
R2 or M1** (n=656)	237 (36.1%)	64 (9.8%)	48 (7.3%)	349 (53.2%)
**Type 2 DM (126,639**)				
R0 M0 (n=92,543)	942 (1.0%)	73 (0.1%)	82 (0.1%)	1,097 (1.2%)
R1 (one eye) M0 (n=20,147)	822 (4.1%)	62 (0.3%)	45 (0.2%)	929 (4.6%)
R1 (both eyes) M0** (n=11,978)	1,663 (13.9%)	264 (2.2%)	128 (1.1%)	2,055 (17.2%)
R2 or M1** (n=1,971)	530 (26.9%)	184 (9.3%)	60 (3.0%)	774 (39.3%)

*data available for those screened prior to 2009 and with at least 3 years of follow-up; therefore, these numbers do not accurately reflect numbers available for recruitment (45% greater at last count)

**patient groups to be recruited in LENS; proportional recruitment from each of these groups provides 29% progression over 4 years

6.1.1 Planned recruitment rate

It is estimated that there are >20,000 patients in Scotland meeting the DR eligibility criteria for LENS. It will be necessary to recruit approximately 5% of these individuals. Due to the opportunity to perform preliminary screening before meeting potential participants, it is anticipated that face to face screening will be highly efficient. The use of an active run-in phase is designed to include individuals who are likely to remain adherent to trial procedures, thereby maximising the trial's ability to detect a true effect of the study treatment. We anticipate 25% loss of patients during the screening and active run-in phases combined. Approximate total recruitment targets for randomised participants in each health board site are listed below, and these are determined by population.

NHS Greater Glasgow and Clyde	245
NHS Forth Valley	62
NHS Lothian	170
NHS Grampian	115
NHS Lanarkshire	114
NHS Tayside	84
NHS Fife	75
NHS Ayrshire and Arran	75
NHS Highland	64
NHS Dumfries and Galloway	32
NHS Borders	24
TOTAL	*1060*

Using the opening of the first site for screening visits as Month 0, we aim to recruit approximately the following numbers: 69 participants at 6 months, 368 at 12 months, 729 at 18 months and 1060 at 24 months from the start of recruitment. These estimates are based on opening all sites over a seven month period and aiming to open sites in the largest health boards first, with modest recruitment for the first two months followed by stable recruitment until the end of the recruitment period.

6.2 STATISTICAL ANALYSIS

The trial data will be held in a secure database in the CCO with controlled access in line with departmental security policies. Analyses will be performed in accordance with a pre-specified Statistical Analysis Plan (SAP), which will be written by the trial statisticians and approved by the TSC prior to any unblinding of study results, and which will describe in detail the methods used to analyse each outcome and the approaches used to handle any missing data. All analyses will be done according to the “intention-to-treat” principle (i.e. participants will be analysed in the group to which they were randomised, no matter what treatment they received, and regardless of whether they deviated from the protocol in any way). The primary analysis of the primary outcome will be considered statistically significant at the 5% significance level. For secondary and, particularly, tertiary outcomes, and also for secondary analyses of the primary outcome, allowance in their interpretation will be made for multiple hypothesis testing, taking into account the nature of events (including timing, duration and severity) and evidence from other studies. In addition to the pre-specified comparisons, other analyses may be performed with due allowance for their exploratory and, perhaps, data-dependent nature.

6.2.1 Interim analysis and criteria for the premature termination of the trial

Unblinded interim analyses will be reviewed annually by the DMC, and will be conducted and presented by a statistician not involved in the day-to-day management of the trial. Upon review of such data, the DMC will advise the Steering Committee if, in their view, the randomised comparisons have provided both (a) proof beyond reasonable doubt that for all patients, or for some specific types, fenofibrate therapy is clearly indicated or contra-indicated; and (b) evidence that might reasonably be expected to influence materially the patient management of many clinicians. The TSC can then decide whether to end or modify the trial or to seek additional data, and this decision will be communicated in writing to the CI after each TSC meeting. Only members of the DMC will have access to unblinded results during the conduct of the trial. Any decision by the DMC which is deemed relevant to the safety of participants will be reported in an expedited fashion to the MHRA.

Appropriate criteria of proof beyond reasonable doubt cannot be specified precisely, but the DMC will work on the principle that a difference of at least 3 standard errors in an interim analysis of a major outcome event may be needed to justify halting, or modifying, a study before the planned completed recruitment. This criterion has the practical advantage that the exact number of interim analyses would be of little importance, and so no fixed schedule is proposed[41].

6.2.2 Economic evaluation

An economic evaluation will be carried out alongside the trial, adopting a health service perspective on costs and a patient perspective on health benefits. The cost of providing the study drug over and above standard care will be estimated based on drug prescription and administration costs. Any increase in primary care monitoring (e.g. blood and urine testing) will be captured from the SCI Diabetes system. All secondary care health service resource use will be captured by electronic linkage to routine datasets: SMR01 for all inpatient and day case activity; SMR00 for all outpatient contacts (http://www.ndc.scot.nhs.uk/National-Datasets/index.asp). DR specific referrals and procedures will be captured using a combination of cross checked sources: the SCI diabetes system; relevant OPCS codes from SMR01 (SMR00 for ophthalmology outpatient referral and monitoring visits); and the trial specific questionnaire (with verification of reported events by a research nurse cross checking against NHS records). All resource use events will be valued using appropriate national unit prices. The study drug will be costed at list price (by dose and quantity) as reported in most up to data version of the BNF (2015). DR related procedures will be costed where possible using the Scottish national tariff for the relevant health care resource grouping (http://www.isdscotland.org/Health-Topics/Finance/Scottish-National-Tariff/), whilst other inpatient and outpatient care will be costed using Scottish specialty unit cost data (ISD 2015). Primary care contacts will be costed using Personal Social Services Research Unit cost data (2015), and resultant biochemistry tests will costed based on ISD reported unit costs for relevant laboratory services (ISD 2015). Following compilation of the cost dataset, the total health service cost will be estimated for each participant from baseline through to 4 years post randomisation.

Analysis of the economic data will be performed by intention to treat in accordance with a pre-specified economic analysis plan (to be completed before unblinding of the data). The mean difference in total service costs between treatment allocation groups will be estimated using generalised linear regression adjusted for minimisation and baseline covariates (e.g. baseline health status) as appropriate. Costs will be assessed against the primary outcome, and the incremental cost per progression of DR (or DR related intervention) averted will be estimated for fenofibrate versus placebo. In addition, participant responses to the EQ-5D, at baseline and follow-up, will be used to estimate QALYs for each participant. The mean difference in QALYs between the groups will be estimated using generalised linear regression, allowing the incremental cost-per QALY to be estimated for fenofibrate versus placebo based on data collected to 4 years.

If the use of fenofibrate is found to result in an increased cost to the health service at four years, for a modest reduction in progression and a QALY gain of insufficient size to justify the extra cost, modelling will be used to extrapolate costs and QALYs beyond the follow-up period of the trial. This will rely on the fitting of parametric survival curves to the observed progression data, to predict progression beyond four years. Further downstream treatment/monitoring costs (associated with progression), and visual acuity and quality of life outcomes of referable disease, will then be modelled using a combination of data collected within the trial and published data on expected outcomes of treatment. This will allow longer-term cost-effectiveness, in terms of the incremental cost per QALY gained, to be estimated for fenofibrate versus placebo. The robustness of any

model based estimates of cost-effectiveness will be assessed using deterministic and probabilistic sensitivity analysis.

7 DATA HANDLING

A web-based eCRF and dedicated TMS will be used to collect study data from the screening and randomisation visits as well as for recording subsequent follow-up assessments of participants every six months. The follow-up assessments (questionnaires) will be conducted as a telephone interview by the LCC nurse, or by review of medical records where necessary. All data in the study will be processed electronically using a set of custom-written applications. CCO staff will use a suite of administration applications to manage sites and study participants, including clinical supervision (review of reported SAEs and routinely collected blood results), management of follow-up and compliance, collection of supporting documentation for relevant events and event adjudication (where relevant). All data will be stored securely. All data accesses will require a unique username and password, and any changes to data will require the user to enter their username and password as an electronic signature. Staff will have access restricted to only the functionality and data that are appropriate to their role in the study. Possible errors or omissions in the completion of eCRFs by research staff will result either in immediate queries (e.g. if pre-set boundaries are exceeded) or subsequent data queries. Errors or omissions in the completion of forms by participants will result, if appropriate, in requests to participants for clarification. Procedures for determining if changes to recorded data are appropriate will be developed and implemented prior to unblinding of the study results. All changes to the data will result in an audit trail. The CCO is also responsible for seeking information regarding pre-specified outcomes, deaths and hospitalisations via linkage to data registries.

7.1 SOURCE DATA

Data recorded in the eCRF from face-to-face visits with participants, lists of potentially eligible patients provided by SDRN and known through responses to DES mail-out and HIC mail-out invitations (to be stored within respective LCCs and/or the CCO), electronically completed questionnaire data, information obtained from telephone calls to the Freephone number regarding the study, information obtained via electronic linkage to central registries regarding pre-specified outcomes and other events, and biochemistry results, pharmacy records (from the commercial partner who will package, store, select and send out study drug by routine post) and documents collected regarding medical events of interest (including copies of information regarding eye procedures) will constitute the ‘source documents’ for the trial. The CCO will retain these data and records for at least 25 years (identifiable data for 10 years) after the end of the trial except in the case of any documentation which is legally required to be retained in Scotland in which case such documents will be stored at the University of Glasgow under the same conditions. Regulatory Authorities will have the right, in accordance with Good Clinical Practice (GCP) guidelines, to commission a confidential audit of such records while the trial is in progress or thereafter.

7.2 ACCESS TO DATA

Records will be kept in a secure storage area with limited access. CTSU is a secure building with access limited to employees and authorised visitors. The study servers are located in a climate controlled secure enclosure to which only system support staff have access. Direct access will be granted to authorised representatives from the Sponsor, host institution and the regulatory authorities to permit trial-related monitoring, audits and inspections.

7.3 DATA PROTECTION AND PATIENT CONFIDENTIALITY

All investigators and trial site staff will comply with the requirements of the United Kingdom General Data Protection Regulation (UK-GDPR) and Data Protection Act 2018 with regards to the collection, storage, processing and disclosure of personal information. At baseline, identifiable data will be recorded as detailed in section 4.5.1 by the LCCs. This will include the CHI number which is crucial for performing electronic linkages with relevant registries. Identifiable details will be stored in a Contact Database in encrypted format with controlled access for research staff in line with departmental security policies. Participants will be identified throughout by means of a unique participant ID number made up of an unrelated sequence of numbers. Local sites will securely store source data including ICFs, biochemistry results, and information regarding any events of interest and these documents will have identifiable data on them. Questionnaire data will be collected electronically, processed and securely stored at the CCO.

Linkage to health registries is entirely dependent on use of each participant's CHI number and data will be collected using pseudonymisation. For the purposes of data linkage, named colleagues in each registry (e.g. eDRIS, SCI Diabetes, DES) will be sent a list of CHI numbers and other identifiable data that they require for participants along with a unique trial identifier by the CCO. Data returned to the CCO will include the relevant results and/or outcomes along with the unique trial identifiers but no identifiable characteristics. The data will be incorporated into the TMS. Data transmitted for any regulatory purposes will contain no identifiable data. PBPP approval is required for linkage to Scottish health registries and this will be required prior to any linkage.

7.4 ACCESS TO THE FINAL TRIAL DATASET

The CCO Investigators and statisticians will have full access to the study data-set. Data generated by LENS will be available to bona fide researchers via the Richard Doll Centenary Archive (see https://www.ndph.ox.ac.uk/about/richard-doll-centenary-archive) which has a Data Access and Sharing Policy.

7.5 ARCHIVING

The CCO will retain trial data and records for at least 25 years (identifiable data for 10 years) after the end of the trial except in the case of any documentation which is legally required to be retained in Scotland in which case such documents will be stored at the University of Glasgow under the required conditions. Regulatory Authorities will have the right, in accordance with GCP guidelines, to commission a confidential audit of such records while the trial is in progress or thereafter.

8 MONITORING, AUDIT & INSPECTION

The trial will be conducted in accordance with the principles of the International Conference on Harmonisation Guidelines for GCP, and relevant local, national and international regulations (including the EU Clinical Trials Directive).

Monitoring will be based on the study's risk assessment and will be detailed in the study Monitoring Plan. The Monitoring Plan will be agreed by the Trial Management Group (TMG) and TSC. The Monitoring Plan will consist of both central monitoring and on site monitoring, both of which will be conducted and overseen by the CCO.

Prior to the initiation of the study at any site, it will be verified that the LCC has adequate facilities and resources to carry out the study (and, if considered necessary, a site visit will be undertaken). Local PIs and Research Nurses will be provided with materials detailing relevant study procedures, and the LCC will be trained in study methods.

Monitoring visits at LCCs are intended at the following stages: around site set-up, by the end of month 12, by the end of month 30, and around month 48 in preparation for site closure. If, an on-site visit cannot be accommodated at approximately these time points, the site will be asked to complete a review of their Investigator Site File and confirm completeness. During on-site visits, the LCC staff are expected to be present. Where an on-site visit coincides with a participant study visit, the monitor may be present during the interview (subject to the participant's verbal agreement) to provide ongoing training in study procedures such as consent taking and the use of the study eCRF. The purpose of site visits is to help LCC staff to resolve any local problems with the study, to ensure that the study is conducted according to the protocol, and to review study records and data quality and provide any additional training that may be required. A report of each visit will be prepared by the study monitor and reviewed by CCO staff.

Central monitoring of the study data will be performed to confirm data completeness, timely entry and to identify any outliers in relation to study matrix e.g. recruitment rates, reporting of events. This central monitoring will be used to direct any non-routine site visits.

If any party becomes aware of an upcoming audit or inspection, the monitoring team at the CCO should be informed. The CCO will advise what preparation may be required and will suggest appropriate personnel to support the audit/inspection.

9 ETHICAL AND REGULATORY reports and CONSIDERATIONS

Before the start of the trial, approval will be sought from a REC for the trial protocol, ICFs and other relevant documents. Substantial amendments that require review by REC will not be implemented until the REC grants a favourable opinion (unless failure to do so would introduce risk to participant/s in the opinion of the investigator). Correspondence with the REC will be retained in the Trial Master File and Investigator Site Files (ISF). Annual progress reports will be submitted to the REC within 30 days of the anniversary date on which the favourable opinion was given, and annually until the trial is declared ended. It is the CI's responsibility to produce the annual reports as required and to notify the REC of the end of the study. Within one year after the end of the study, the CI will submit a final report with the results to the REC.

9.1 PEER REVIEW

Prior to submission for funding, the trial proposal was internally reviewed by one expert (Professor John Petrie, University of Glasgow). The full trial application to NIHR underwent peer review by seven independent experts, and the trial's design was modified based on their comments and suggestions.

9.2 PUBLIC AND PATIENT INVOLVEMENT

On 18th March 2015, two LENS investigators met with 15 members of the Glasgow South Diabetes UK Group to present and discuss LENS. The members advised on patient perceptions of the research question and practicalities of delivering the trial. They indicated that they understood the need for a control group. Two patients with diabetes have volunteered to join the TSC as patient representatives and these individuals will be involved with preparing and reviewing study information and providing patient perspectives on the trial's conduct. The Royal National Institute for the Blind (RNIB), Scotland, is also supporting the trial in terms of dissemination of information and results of the trial, review of materials, lay summaries and newsletters, and representation on the TSC. Feedback regarding the various PILs to be used in the trial and the content of

questionnaires will be sought from the Glasgow South Diabetes UK Group and/or other patient support groups.

9.3 AMENDMENTS

The sponsor may make a non-substantial amendment at any time during a trial. If the sponsor wishes to make a substantial amendment to the CTA or the documents that supported the original application for the CTA, the sponsor will submit a valid notice of amendment to the MHRA. If the sponsor wishes to make a substantial amendment to the REC application or the supporting documents, the sponsor must submit a valid notice of amendment to the REC for consideration. It is the sponsor’s responsibility to decide whether an amendment is substantial or non-substantial. Amendments will be notified to NHS Research & Development departments of participating sites to assess whether the amendment affects the NHS permission for that site. The CI will be responsible for the decision to amend the protocol.

9.4 COMPLIANCE

9.4.1 Protocol compliance

Protocol deviations, violations, or breaches are departures from the approved protocol. Prospective, planned deviations or waivers to the protocol are not allowed under the UK regulations on Clinical Trials and must not be used. Accidental protocol deviations must be documented and reported to the CI.

9.4.2 Regulatory Compliance

The trial will not commence until a Clinical Trial Authorisation (CTA) is obtained from the MHRA. Before any site can enrol patients into the trial, the local PI or designee will apply for and obtain permission from the site’s NHS Research & Development (R&D) department.

9.4.3 Notification of Serious Breaches to GCP and/or the protocol

A “serious breach” is a breach which is likely to affect to a significant degree the safety or physical or mental integrity of the subjects of the trial; or the scientific value of the trial.

In the event that a serious breach is suspected, the CCO and CI must be contacted within 1 working day. It is the CI’s responsibility to contact the Sponsor without delay. In collaboration with the CI, the serious breach will be reviewed by the Sponsor and, if appropriate, the Sponsor will report it to the REC committee, Regulatory authority and the NHS host organisation within seven calendar days.

9.5 FINANCIAL AND OTHER COMPETING INTERESTS

The trial investigators and research team have no conflicts of interest regarding the conduct of the trial.

9.6 INDEMNITY

The University of Oxford has a specialist insurance policy in place which would operate in the event of any participant suffering harm as a result of their involvement in the research (Newline Underwriting Management Ltd, at Lloyd’s of London). NHS indemnity operates in respect of the clinical treatment that is provided.

10 DISSEMINATION POLICY

On completion of the trial, the data will be analysed and tabulated and a Final Study Report prepared. Funding organisations will be acknowledged in all resultant publications. Participants will be informed of the main results of the trial with a newsletter or letter. The protocol will be made publically available on the NIHR website during the conduct of the trial and will also be included as a supplementary file at the time of publishing the main trial results.

Draft copies of manuscripts will be circulated to all co-investigators and local PI for their review prior to submission for publication. Results from the trial will be published in the name of the LENS collaboration.

The design paper, primary LENS results publication and subsequent publications will be submitted for publication in medical journals in an open access format. RNIB will cover the trial results on their webpage and via other strategies. The results will be submitted for presentation at appropriate conferences.

11 REFERENCES

1. Ciulla TA, Amador AG, Zinman B. Diabetic retinopathy and diabetic macular edema: pathophysiology, screening, and novel therapies. *Diabetes Care* 2003; **26**(9): 2653-64.
2. Liew G, Michaelides M, Bunce C. A comparison of the causes of blindness certifications in England and Wales in working age adults (16-64 years), 1999-2000 with 2009-2010. *BMJ Open* 2014; **4**(2): e004015.
3. Looker HC, Nyangoma SO, Cromie DT, et al. Rates of referable eye disease in the Scottish National Diabetic Retinopathy Screening Programme. *Br J Ophthalmol* 2014; **98**(6): 790-5.
4. Watkins PJ. Retinopathy. *BMJ* 2003; **326**(7395): 924-6.
5. Luckie R, Leese G, McAlpine R, et al. Fear of visual loss in patients with diabetes: results of the prevalence of diabetic eye disease in Tayside, Scotland (P-DETS) study. *Diabet Med* 2007; **24**(10): 1086-92.
6. Coyne KS, Margolis MK, Kennedy-Martin T, et al. The impact of diabetic retinopathy: perspectives from patient focus groups. *Fam Pract* 2004; **21**(4): 447-53.
7. UK Prospective Diabetes Study (UKPDS) Group. Intensive blood-glucose control with sulphonylureas or insulin compared with conventional treatment and risk of complications in patients with type 2 diabetes (UKPDS 33). *Lancet* 1998; **352**(9131): 837-53.
8. The Diabetes Control and Complications Trial/Epidemiology of Diabetes Interventions and Complications Research Group. Retinopathy and nephropathy in patients with type 1 diabetes four years after a trial of intensive therapy. *N Engl J Med* 2000; **342**(6): 381-9.
9. Boussageon R, Bejan-Angoulvant T, Saadatian-Elahi M, et al. Effect of intensive glucose lowering treatment on all cause mortality, cardiovascular death, and microvascular events in type 2 diabetes: meta-analysis of randomised controlled trials. *BMJ* 2011; **343**: d4169.
10. Duncan LJ, Cullen JF, Ireland JT, Nolan J, Clarke BF, Oliver MF. A three-year trial of atromid therapy in exudative diabetic retinopathy. *Diabetes* 1968; **17**(7): 458-67.
11. Staels B, Dallongeville J, Auwerx J, Schoonjans K, Leitersdorf E, Fruchart JC. Mechanism of action of fibrates on lipid and lipoprotein metabolism. *Circulation* 1998; **98**(19): 2088-93.
12. Keech A, Simes RJ, Barter P, et al. Effects of long-term fenofibrate therapy on cardiovascular events in 9795 people with type 2 diabetes mellitus (the FIELD study): randomised controlled trial. *Lancet* 2005; **366**(9500): 1849-61.
13. The ACCORD Study Group. Effects of combination lipid therapy in type 2 diabetes mellitus. *N Engl J Med* 2010; **362**(17): 1563-74.
14. Keech AC, Mitchell P, Summanen PA, et al. Effect of fenofibrate on the need for laser treatment for diabetic retinopathy (FIELD study): a randomised controlled trial. *Lancet* 2007; **370**(9600): 1687-97.
15. The ACCORD Study Group and ACCORD Eye Study Group. Effects of medical therapies on retinopathy progression in type 2 diabetes. *N Engl J Med* 2010; **363**(3): 233-44.
16. Chen Y, Hu Y, Lin M, et al. Therapeutic effects of PPARalpha agonists on diabetic retinopathy in type 1 diabetes models. *Diabetes* 2013; **62**(1): 261-72.
17. Royal College of Ophthalmologists. Diabetic Retinopathy Guidelines. 2012. https://www.rcophth.ac.uk/wp-content/uploads/2021/08/2012-SCI-267-Diabetic-Retinopathy-Guidelines-December-2012.pdf. (last accessed 9 June 2022)
18. National Institute for Health and Care Excellence. Type 2 diabetes. 2008. http://www.nice.org.uk/guidance/cg66. (last accessed 9 June 2022)
19. Scottish Intercollegiate Guidelines Network. Management of Diabetes. 2010. https://www.sign.ac.uk/assets/sign116.pdf. (last accessed 9 June 2022)
20. FAME 1 EYE. 2011. http://clinicaltrials.gov/show/NCT01320345. (last accessed 9 June 2022)
21. Aung T, Haynes R, Barton J, et al. Cost-effective recruitment methods for a large randomised trial in people with diabetes: A Study of Cardiovascular Events iN Diabetes (ASCEND). *Trials* 2016; **17**(1): 286.
22. Preiss D, Lloyd SM, Ford I, et al. Metformin for non-diabetic patients with coronary heart disease (the CAMERA study): a randomised controlled trial. *Lancet Diabetes Endocrinol* 2014; **2**(2): 116-24.
23. Scottish Diabetes Research Network. 2015. http://www.sdrn.org.uk/. (last accessed 9 June 2022)
24. NHS Research Scotland. SHARE. 2015.
25. Scottish Care Information - Diabetes Collaboration. 2015.
26. Kostapanos MS, Florentin M, Elisaf MS. Fenofibrate and the kidney: an overview. *Eur J Clin Invest* 2013; **43**(5): 522-31.

27. British National Formulary. Fenofibrate. 2015. https://www.medicinescomplete.com/mc/bnf/current/PHP1686-fenofibrate.htm. (last accessed 9 June 2022)
28. EuroQol. EQ-5D. 2016.
29. National Eye Institute. National Eye Institute Visual Function Questionnaire. 2016.
30. Sauron R, Wilkins M, Jessent V, Dubois A, Maillot C, Weil A. Absence of a food effect with a 145 mg nanoparticle fenofibrate tablet formulation. *Int J Clin Pharmacol Ther* 2006; **44**(2): 64-70.
31. Jun M, Zhu B, Tonelli M, et al. Effects of fibrates in kidney disease: a systematic review and meta-analysis. *J Am Coll Cardiol* 2012; **60**(20): 2061-71.
32. Mychaleckyj JC, Craven T, Nayak U, et al. Reversibility of fenofibrate therapy-induced renal function impairment in ACCORD type 2 diabetic participants. *Diabetes Care* 2012; **35**(5): 1008-14.
33. Davis TM, Ting R, Best JD, et al. Effects of fenofibrate on renal function in patients with type 2 diabetes mellitus: the Fenofibrate Intervention and Event Lowering in Diabetes (FIELD) Study. *Diabetologia* 2011; **54**(2): 280-90.
34. Bonds DE, Craven TE, Buse J, et al. Fenofibrate-associated changes in renal function and relationship to clinical outcomes among individuals with type 2 diabetes: the Action to Control Cardiovascular Risk in Diabetes (ACCORD) experience. *Diabetologia* 2012; **55**(6): 1641-50.
35. Scottish Diabetes Survey Monitoring Group. Scottish Diabetes Survey 2014. 2014. https://www.diabetesinscotland.org.uk/wp-content/uploads/2019/12/Diabetes-in-Scotland-website-Scottish-Diabetes-Survey-2014.pdf. (last accessed 9 June 2022)
36. Linz PE, Lovato LC, Byington RP, et al. Paradoxical reduction in HDL-C with fenofibrate and thiazolidinedione therapy in type 2 diabetes: the ACCORD Lipid Trial. *Diabetes Care* 2014; **37**(3): 686-93.
37. Whitfield LR, Porcari AR, Alvey C, Abel R, Bullen W, Hartman D. Effect of gemfibrozil and fenofibrate on the pharmacokinetics of atorvastatin. *J Clin Pharmacol* 2011; **51**(3): 378-88.
38. Law M, Rudnicka AR. Statin safety: a systematic review. *Am J Cardiol* 2006; **97**(8A): 52C-60C.
39. Guo J, Meng F, Ma N, et al. Meta-analysis of safety of the coadministration of statin with fenofibrate in patients with combined hyperlipidemia. *Am J Cardiol* 2012; **110**(9): 1296-301.
40. Bergman AJ, Murphy G, Burke J, et al. Simvastatin does not have a clinically significant pharmacokinetic interaction with fenofibrate in humans. *J Clin Pharmacol* 2004; **44**(9): 1054-62.
41. Peto R, Pike MC, Armitage P, et al. Design and analysis of randomized clinical trials requiring prolonged observation of each patient. I. Introduction and design. *Br J Cancer* 1976; **34**(6): 585-612.

12 APPENDICES

12.1 APPENDIX 1 - RISK ASSESSMENT

A risk assessment of the trial was performed prior to final sign-off of the contract with the funder. This risk assessment is a controlled version working document which is subject to change. It is stored in EDMS, the document management system of CTSU. The risk assessment will form the basis of the Trial Monitoring Plan.

12.2 APPENDIX 2 – AUTHORISATION OF LOCAL COLLABORATING CENTRES

12.2.1 Required documentation

Prior to opening a LCC, the following essential documentation will be provided to the sponsor:

- Evidence of local R&D approval
- An original signed Investigator Declaration (signed by the local PI as part of Clinical Trial Agreement documentation)
- A signed and dated recent CV for the local PI and research nurse
- Copies of GCP certificates for the local PI and research nurse

12.2.2 Procedure for initiating/opening a new site

Trial staff from the RCC (or from the CCO where this is not possible) will perform training for the local PI, research nurse and any other staff involved in the trial at the LCC.

12.2.3 PI responsibilities

The local PI at each site is responsible for the overall conduct of the study at their site and for compliance with the protocol and any protocol amendments. Responsibilities may, however, be delegated to an appropriate member of study site staff. Delegated tasks will be documented on a Delegation Log and signed by all those named on the list. The local PI must be familiar with the IMP, protocol and the study requirements.

12.3 APPENDIX 3 – SCHEDULE OF ASSESSMENTS

PROCEDURE	Screening Visit	Randomisation visit	Subsequent telephone / email follow-up (every 6 months for at least 3 years unless stated otherwise)
Informed consent	X		
Inclusion / exclusion criteria	X	X	
Record demographics	X	X	
Medical History	X	X	
Lifestyle factors	X		
Concomitant medications	X	X	
Height		X	
Weight		X	
Vital signs		X	
Blood eligibility biochemistry	X[a]	X[a]	
Blood HbA1c	X		
Blood lipids (random)	X		
Urine albumin creatinine ratio	X		
Trial-specific Questionnaire (by telephone or medical records)			X
EQ-5D questionnaire (on paper)	X		X[d]
VFQ-25 questionnaire (on paper)	X		X[d]
Open-label fenofibrate mail-out (10 week supply)	X[b]		
Blinded drug mail-out (26 week supply)		X	X
Compliance with study treatment		X	X
Randomisation		X[c]	
Willingness to start and/or continue study treatment	X	X	X

[a] U&E, LFTs (including at least one of ALT and AST), CK

[b] run-in drug mail-out will not be immediate as it will first be necessary to verify biochemistry; the initial mail-out of open-label fenofibrate will be followed by a phone call 2-3 weeks later to verify receipt by the participant

[c] randomisation will not occur at the time of the visit as it depends on visit biochemistry; starting dose frequency will depend on renal function

[d] for completion after 24 months and then at the final assessment

12.4 APPENDIX 4 – PROTOCOL AMENDMENT HISTORY

**Substantial / non-substantial amendment**	**Protocol version no.**	**Date issued**	**Author(s) of changes**	**Details of changes made**
Approved by REC	2.0	21-09-2016	D. Preiss<br>S. Howard	• Section 4.8: removal of need to provide written confirmation of withdrawal<br>• Section 3.2.3: expansion of potential exploratory endpoints for OCT<br>• Minor clarifications or corrections to text on pages 27, 29, 32, 35, 47, 56
Substantial amendment 1	3.0	26-04-2017	D. Preiss<br>S.Howard	• Additional method of recruitment – via HIC<br>• Revision of section 5 –Safety reporting<br>• Change from registered to routine postal delivery of study treatment<br>• Clarifications or corrections to text throughout the document
Substantial amendment 2	4.0	07-07-2017	D.Preiss<br>S. Howard	• Revised wording in section 4.4.4 to clarify procedural steps for recruitment using HIC services.
Substantial amendment 3	5.0	05-10-2017	D. Preiss<br>C. Knott<br>S. Howard	• P.3 Key Trial Contacts – addition of Dr R Lindsay<br>• Section 3.2 – movement of text<br>• Section 4.4.2 – addition of a telephone call to potential participants to ask if they would be interested in receiving information about LENS and seek verbal consent to check key eligibility criteria in their health records (specifically their most recent eGFR result and retinal screening results).<br>• Section 4.5.1 – correction of 6-10 to 6-11 weeks<br>• Section 4.11 – updating Manufacturer's Authorisation; inclusion of plan to provide low HDL-c data to DMC<br>• Section 5.3 – clarification regarding AE reporting
Substantial amendment 4	6.0	10-01-2018	D. Preiss<br>S.Howard	Changes added based on MHRA review:<br>• Section 4.11.12 – Clarification regarding true abstinence

				• Sections 4.2.2, 4.11.10, 4.11.11 – Exclusion criterion added: rosuvastatin 40mg daily<br>• Section 4.5.10 – Refinement of creatine kinase assessment prior to randomisation<br>• Section 4.6.6 – Clarification regarding unblinding<br>• Section 4.8.1 – Addition of list of circumstances which will lead to cessation of study treatment<br>• Section 5.3.3 – Clarification regarding reporting urgent safety measures
Non-substantial amendment 2	6.1	12-02-2019	D. Preiss<br>S. Howard	Minor edits:<br>Addition of collaborators (p4)<br>Exclusion criteria clarified to make distinct the biochemical eligibility for each face-to-face assessment visit and for lack of adherence to run-in treatment (4.2.2)<br>Clarification regarding timing of call to check receipt of run-in drug (4.5.3)
Non-substantial amendment 4	6.2	29-05-2020	D. Preiss	Remove treatment duration (summary)<br>Clarify minimum trial duration (summary; section 2)<br>Allow for DRS grading to not change (footnote to Table 1)<br>Clarify that evidence of clinically significant DR may come from retinal imaging or clinical examination (3.2.1, Table 2)<br>Adjudicate vitreous haemorrhage (3.3)<br>Remove comment on discrepancy between data sources (3.3)
Non-substantial amendment 5	6.3	06-01-2021	D. Preiss	Participants will not complete follow up questionnaires on their own devices; follow up by medical records to be used where necessary (4.6.1, 4.7, 5.3.1, 7, 12.3)<br>EQ-5D and VFQ-25 will only be completed on paper (4.5.1, 4.7, Table 3, 12.3)<br>Source data to be retained for 25 years, but identifiable data for 10 years after trial (7.1, 7.5)

				Clarify description of introduction of OCT by NHS Scotland (Table 1 and footnote)<br>Update site monitoring plan in context of COVID pandemic
Substantial amendment 5	7.0	29-07-2022	D. Preiss	Remove signature page (protocol is signed off in the EDMS system)<br>Change Jen Logue details (page 3)<br>Remove Enti Spata (page 3)<br>Include details of change in NHS Scotland management pathway for M2 maculopathy: Section 1, Table 1<br>Use consistent wording to describe M2, R3 and R4 as ‘referable’ disease (rather than ‘clinically significant’ or ‘significant’ disease): Trial summary, Sections 3, 4.2 and 6.1; Table 2<br>Diabetic Retinal Screening (DRS) update to Diabetic Eye Screening (DES) after NHS Scotland change to program name: various Sections<br>DES definition for blot (vs. dot) haemorrhage added: Table 1<br>Clarification that M2 includes blot haemorrhages (which has always been the case): Trial summary, Sections 3.1.2, 3.2.2; Table 2<br>Include plans for use of image analysis software at a later stage: Section 3.2<br>Allow for future meta-analyses of all fenofibrate trials: Section 3.2.4<br>Minor grammatical correction 4.11.13<br>Shorten heading Section 5.3<br>Correct reference to UK GDPR and Data Protection Act 2018: Section 7.3<br>It was not possible to offer the option for participants to complete follow up forms on their own devices, line removed: Section 4.5.1.<br>Show details of amendment numbers in Appendix 4

A randomised placebo-controlled trial of fenofibrate to prevent progression of non-proliferative retinopathy in diabetes

(Lowering Events in Non-proliferative retinopathy in Scotland [LENS])

Statistical Analysis Plan

EDMS #7695

Version 1.3

Table of Contents

1 VERSION HISTORY 3

2 TRIAL REGISTRATION DETAILS 4

3 INTRODUCTION 5

3.1 STUDY DESIGN 5
3.2 STUDY OBJECTIVES 5

4 DEFINITIONS FOR EFFICACY AND SAFETY ENDPOINTS 6

4.1 ESTIMANDS 6
4.2 HYPOTHESES 6
4.3 PRIMARY EFFICACY ASSESSMENT 6
4.4 SECONDARY EFFICACY ASSESSMENTS 6
4.4.1 Secondary assessments based on clinical events 6
4.4.2 Secondary assessments based on questionnaires and visual acuity 7
4.4.3 Health economic assessments 7
4.5 TERTIARY EFFICACY ASSESSMENTS 7
4.5.1 Tertiary assessments based on clinical events 7
4.5.2 Tertiary assessments based on biochemistry 7
4.6 CLINICAL SAFETY ASSESSMENTS 7
4.7 OTHER LABORATORY ASSESSMENTS 8

5 ANALYSIS SETS FOR EFFICACY AND SAFETY ANALYSES 8

6 ASCERTAINMENT AND ADJUDICATION OF STUDY ENDPOINTS 8

6.1 ASCERTAINMENT OF INFORMATION 8
6.2 ADJUDICATION 9

7 STATISTICAL METHODOLOGY FOR EFFICACY AND SAFETY ANALYSES 9

7.1 METHODS OF ANALYSIS 9
7.2 MULTIPLICITY ADJUSTMENTS 10
7.3 SUBGROUP ANALYSES AND TESTS FOR HETEROGENEITY 10
7.4 HANDLING OF MISSING AND INCOMPLETE DATA 10
7.5 CENSORING SCHEMA FOR TIME-TO-EVENT ENDPOINTS 11

8 GUIDELINES FOR EARLY STOPPING OR MODIFICATION OF THE TRIAL 11

9 REFERENCES 12

10 APPENDIX A: DETAILS OF RANDOMISATION ALGORITHM 13

1 Version History

0.1	Draft version	29 November 2021
0.2	Updated after SC comments	20 April 2022
1.0	First released version	29 June 2022
1.1	Minor clarifications to Section 5	10 January 2023
1.2	Exact subgroup categories now defined and description of MMRM models for continuous measures measured at multiple follow-up time points. Report frequency of NHS retinal screening. Add exploratory primary outcome analysis excluding first 12 months. Clarify visual acuity to be analysed as LogMAR (with conversion from Snellen where required).	12 June 2023
1.3	Appendix A: smoking was never included as minimisation variable, SAP error corrected	07 February 2024

2 Trial registration details

IRAS Number:	209579
EudraCT Number:	2016-002656-24
Clinical trials.gov Number:	NCT03439345
ISRCTN Number	ISRCTN15073006
REC Number:	16/WS/0149
Sponsor’s number:	CTSULENS1
NIHR Number:	14/49/84

3 Introduction

This Statistical Analysis Plan describes the strategy, rationale and statistical methods that will guide assessment of the clinical efficacy and safety of fenofibrate in the LENS trial.

3.1 Study design

LENS is a randomised placebo-controlled trial investigating the effects of prolonged treatment with the PPAR alpha agonist, fenofibrate on the progression of diabetic eye disease. At least 1,060 participants with observable diabetic retinopathy or maculopathy will be randomised (using a minimisation algorithm, see Appendix A: Details of randomisation algorithm) between oral fenofibrate 145mg or placebo tablets. Study treatment is taken once daily in the context of normal renal function, or as one tablet every second day in the context of reduced renal function. Follow up is scheduled to continue until at least 222 primary outcome events have occurred and median follow up (counting from the date of randomisation of the median participant) is at least 4 years.

The trial is designed to be streamlined. Most data for pre-specified efficacy outcomes is obtained by linkage to NHS Scotland records. Only the Screening and Randomisation assessments occur in person, and all subsequent assessments occur by telephone or by reference to medical records. Study treatment is mailed to participants' homes. After the initial Screening visit is conducted and safety blood tests are performed, eligible individuals (who are approved to enter the run-in by the local investigator) are provided with active fenofibrate 145mg tablets to be taken (daily with normal renal function, one tablet every second day with reduced renal function) for approximately 8 weeks. They are then asked to return to the study clinic for a Randomisation assessment. If they remain eligible based on information collected at this assessment and further safety blood tests, and are willing to continue, participants are randomly allocated to fenofibrate 145mg tablets or placebo.

No research blood or urine samples are taken from randomised participants. Linkage to NHS Scotland data-sets is performed to obtain results for HbA1c, renal function (serum creatinine, eGFR, urine albumin, urine albumin creatinine ratio) and other assays (e.g. lipids) that have been collected as part of routine care.

3.2 Study objectives

The primary objective of the LENS trial is to assess the effect of allocation to fenofibrate versus placebo on progression to the composite primary outcome of referable diabetic retinopathy (DR) or any of the following treatments for diabetic retinopathy: retinal laser therapy, vitrectomy or intra-vitreal injections (see Section 4.3).

Other objectives include assessments of the effects of fenofibrate on secondary outcomes (see Section 4.4), tertiary outcomes (see Sections 4.5) and longer term follow up outcomes (to be covered in a separate analysis plan at a later date).

4 Definitions for efficacy and safety endpoints

4.1 Estimands

For the efficacy and safety endpoints based on clinical events, the estimand of interest will be the rate of first occurrence of the endpoint in the target population allocated fenofibrate relative to the rate of first occurrence of the endpoint in the target population allocated placebo, conditional on the baseline covariates used in minimised randomisation, ignoring any non-fatal intercurrent events, and in the hypothetical absence of death from any cause not included in the efficacy or safety endpoint being assessed.

4.2 Hypotheses

For all statistical tests, the null hypothesis will be that the effect of allocation to fenofibrate on the endpoint of interest in the target population is the same as the effect of allocation to placebo.

4.3 Primary efficacy assessment

The primary assessment will involve an intention-to-treat comparison, among all randomised participants, of the effect of allocation to fenofibrate versus placebo on time to the first occurrence during the scheduled treatment period on the following composite primary outcome: the development of referable diabetic retinopathy[a] or any of the following treatments for diabetic retinopathy: retinal laser therapy, vitrectomy or intra-vitreal injection of medication due to diabetic retinopathy

4.4 Secondary efficacy assessments

4.4.1 Secondary assessments based on clinical events

This will involve intention-to-treat comparisons among all randomised participants of the effects of allocation to fenofibrate versus placebo during the scheduled treatment period on time to the first occurrence of:

4.4.1.1 The composite primary outcome in various subgroups:

- Sex (male vs. female)
- Age at randomisation (< 60 years vs. ≥ 60 years)
- Type of diabetes (Type 1 diabetes vs. Type 2 diabetes and other types)
- Renal function (randomisation assessment eGFR[b] < 60 vs. ≥ 60 mL/min/1.73m^2)
- HbA1c (screening assessment HbA1c < 70 mmol/mol vs. HbA1c ≥ 70 mmol/mol)
- First primary outcome in the first year vs subsequent years of follow-up (because of the known reduced impact of lipid modification on cardiovascular events, and potentially reduced impact of fenofibrate on laser treatment for diabetic retinopathy, in the first year of treatment[1,2])

4.4.1.2 The individual components of the composite primary outcome, namely:

- The development of referable diabetic retinopathy[a];
- Treatment for diabetic retinopathy: any of retinal laser therapy, vitrectomy or intra-vitreal injection of medication due to diabetic retinopathy

[a] referable diabetic retinopathy is defined according to NHS Scotland's DES grading as R3 or R4 [excluding R4i as it represents inactive disease] or M2 in at least one eye in the DES grading scheme; such evidence may come from NHS retinal screening or clinical examination

[b] as entered into the eCRF by research staff using whichever formula is employed by that NHS health board

4.4.1.3 Any progression of diabetic retinopathy across the NHS Scotland retinopathy grading scale[c]

4.4.1.4 The presence of hard exudates or blot haemorrhages within 1 disc diameter of the macula[d]

4.4.1.5 Macular oedema

4.4.2 Secondary assessments based on questionnaires and visual acuity

This will involve intention-to-treat comparisons among all randomised participants of the effects of allocation to fenofibrate versus placebo during the scheduled treatment period on:

4.4.2.1 Visual function (based on the Visual Function Questionnaire [VFQ]-25 questionnaire)

4.4.2.2 Quality of life (based on the EuroQol- 5 Dimension [EQ-5D] questionnaire)

4.4.2.3 Visual acuity (based on visual acuity on LogMar or Snellen chart score collected during NHS retinal screening)

4.4.3 Health economic assessments

Health economic assessments will be conducted to help guide the appropriate use of fenofibrate by health care providers. The detailed analysis plan for health economic assessments will be described in a separate document.

4.5 Tertiary efficacy assessments

4.5.1 Tertiary assessments based on clinical events

This will involve intention-to-treat analyses among all randomised participants of the effects of allocation to fenofibrate versus placebo during the scheduled treatment period on time to the first occurrence of:

4.5.1.1 The composite of any major cardiovascular event (defined as myocardial infarction, stroke, coronary or peripheral revascularisation).

4.5.1.2 The composite of any non-traumatic lower limb amputation (defined as minor amputation [distal to the ankle] or major amputation [through or proximal to the ankle]).

4.5.2 Tertiary assessments based on biochemistry

Intention-to-treat analyses of the effects of allocation to fenofibrate versus placebo will be conducted on the following measures:

4.5.2.1 Urine albumin: creatinine ratio

4.6 Clinical safety assessments

SAEs and reasons for stopping study treatment will be captured in the study database to be used for safety assessments. The safety assessments will include intention-to-treat analyses among all randomised participants of the effects of allocation to fenofibrate versus placebo during the scheduled treatment period (with due allowance in their interpretation for multiple comparisons) on:

4.6.1.1 Serious adverse events by Medical Dictionary for Regulatory Activities (MedDRA, version 14.0) System Organ Class classification;

---

[c] progression from R’X’ (baseline) to >R’X’ or from M’X’ (baseline) to >M’X’ in either eye

[d] M2 grading in the NHS Scotland grading scheme

4.6.1.2 Discontinuation of study treatment, overall and by various causes

4.7 Other laboratory assessments

Intention-to-treat analyses of the effects of allocation to fenofibrate versus placebo will be conducted on the following measures:

4.7.1.1 eGFR[e]

4.7.1.2 Lipids (total cholesterol, non-HDL cholesterol, HDL cholesterol, triglycerides)

4.7.1.3 HbA1c

Invitation to routine NHS retinal screening is determined by the most recent retinal screening results (and has also been affected by the COVID-19 pandemic). The mean (standard error) number of retinal screening visits per participant will be calculated for each treatment arm.

5 Analysis sets for efficacy and safety analyses

For all outcomes, analyses will compare the outcome from randomisation to the end of the scheduled treatment period (see Section 7.5) among all those participants who are allocated at randomisation to receive fenofibrate versus all those allocated to receive matching placebo (i.e. “intention-to-treat” analyses).[3,4] Analyses of binary outcomes will be based on the first occurrence of the specified outcome. *Per protocol* analyses will not be conducted because of the potential for bias in such non-randomised assessments.

In accordance with the protocol, primary, secondary and tertiary outcomes will not be adjudicated with the exception of any medical eye procedures, any adverse event reports of vitreous haemorrhage and any adverse event reports of macular oedema. Such events will be adjudicated by clinicians blinded to the study treatment assignment (see Section 6). For those events that are subject to adjudication, analyses will include confirmed and unrefuted events (excluding any events adjudged to be related to a cause other than diabetic retinopathy).

For eye outcome events, the number of people experiencing the outcome (in one or both eyes) will be compared between the treatment groups (rather than the total number of eyes affected).

6 Ascertainment and adjudication of study endpoints

6.1 Ascertainment of information

Unless consent to follow-up is withdrawn, follow-up information is to be collected from all study participants, irrespective of whether or not they continue to receive study treatment. If a participant becomes unwilling or unable to complete follow up study assessments by telephone then study clinic staff will review the available medical records and complete the necessary Follow-up form on the clinic IT system. Information will also be collected from linkage to NHS Scotland healthcare systems and registries. Where retinopathy and maculopathy gradings of retinal images contribute to pre-specified outcomes, the grading results generated by the NHS Scotland Diabetic Eye Screening

[e] Based on calculations using serum creatinine collected by data linkage and the CKD EPI formula:
$eGFR = 141 \times \min(Scr/\kappa, 1)^{\alpha} \times \max(Scr/\kappa, 1)^{-1.209} \times 0.993^{Age} \times 1.018$ [if female] $\times 1.159$ [if African American]
where:
Scr is serum creatinine in µmol/L; κ is 61.9 for females and 79.6 for males; α is -0.329 for females and -0.411 for males; min indicates the minimum of Scr/κ or 1; max indicates the maximum of Scr/κ or 1

program will be used. However, it is also intended to use image analysis software at a later time to repeat these analyses.

6.2 Adjudication

Adjudicators will follow the standardised procedures in accordance with the pre-specified endpoint definitions and adjudication procedures as described in the Adjudication Charter (LENS Adjudication Charter and Outcome Definitions EDMS #6781). Only one blinded adjudicator will execute the adjudication procedure for each event.

7 Statistical methodology for efficacy and safety analyses

7.1 Methods of analysis

All participants randomised to receive fenofibrate will be compared with all participants randomised to placebo, irrespective of whether they received all, some or none of their allocated treatment (i.e. intention-to-treat [ITT] analyses).[3,4]

For the time-to-event analyses, survival analytic methods will be used to evaluate the time to the first event during the entire study period. Cox proportional hazards regression analyses, adjusted for baseline covariates used in minimised randomisation, will be used to estimate the hazard ratios, 95% confidence intervals and corresponding p-values, comparing all participants allocated active fenofibrate with all those allocated placebo.[3] The proportional hazards assumption will be assessed through examination of the Schoenfeld residuals. The Kaplan-Meier estimate will also be plotted for the time to the primary outcome (with its associated log-rank p-value). When separately assessing individual elements of composite outcomes, a participant may contribute to more than one assessment if they have events of more than one type (e.g. significant retinopathy followed by laser treatment). All p-values will be 2-sided. For each of the events listed as additional safety outcomes (Section 4.6), the number of randomised participants with at least 1 event will be compared using standard tests for differences in proportions.

For continuous measures collected only at the screening visit, or which may plausibly be affected by fenofibrate treatment during the active run-in (namely visual function, quality of life, eGFR, lipids [total cholesterol, non-HDL cholesterol, HDL cholesterol, triglycerides], HbA1c, urine albumin: creatinine ratio), the screening visit value will be considered to represent the ‘baseline’ value to which any post-randomisation values are compared. For continuous measures not collected at the screening visit but identified in pre-randomisation NHS linkage data (namely visual acuity), the latest value within 18 months prior to randomisation will be considered to represent the ‘baseline’ value.

Any continuous outcomes that display substantial positive skew will be analysed on the log scale. For quality of life (Section 4.4.2.2), index values will be derived from EQ-5D-5L questionnaire data using a UK-based value set. Visual acuity (Section 4.4.2.3) will be recorded using the LogMAR or Snellen chart score. The main analysis of visual acuity will be based on the eye with best acuity at baseline, and analysis of the eye with worse acuity will be conducted as a sensitivity analysis. Where the baseline visual acuity is the same in both eyes, the right eye shall be chosen for the main analysis and the left eye for the sensitivity analysis. Analyses of visual function will be based on LogMAR values, with conversion of Snellen chart score to LogMAR values where required[f].

Visual function (Section 4.4.2.1) and quality of life (Section 4.4.2.2) are collected at baseline, at approximately 2 years and at the end of the trial as part of scheduled study assessments. By contrast, biochemical measurements (Sections 4.5.2.1 and 4.7) and visual acuity (Section 4.4.2.3) are collected at baseline and then collected at multiple times during follow-up as part of routine NHS clinical care. For all of these outcomes with baseline and repeated follow-up values, linear mixed

[f] LogMAR value = $\log_{10}$(Snellen denominator / Snellen numerator); it is recognised that conversion from Snellen chart score to LogMAR value is imperfect but it is considered adequate for comparison of fenofibrate vs. placebo

model repeated measures (MMRM) analyses will be conducted to estimate the mean values by treatment allocation at each follow-up time point, as well as a trial-averaged difference in mean follow-up levels between the randomised treatment groups. For visual function and quality of life, the relevant follow-up time points will be 2 years and the end of the trial but, for biochemical measurements and visual acuity, the follow-up time points will be the midpoint of each year of follow-up (any individuals with >1 value in a given 1-year post-randomisation window of time will have those values averaged prior to the MMRM analysis). These MMRM models will adjust for the baseline value of the measure of interest as well as the baseline minimisation criteria and will assume an unstructured covariance matrix.

7.2 Multiplicity adjustments

Assuming that the trial runs to its planned completion, the primary assessment at the final analysis will be made first and deemed statistically significant if its two-sided p-value is <0.05.

For the secondary and tertiary assessments, multiple testing will not formally be taken into account. However, given the number of such analyses, due allowance will be made in their interpretation, taking into account the nature of events (including timing, duration and severity) and evidence from other studies.[3,4]

7.3 Subgroup analyses and tests for heterogeneity

The secondary assessments (see Section 4.4.1.1) include intention-to-treat analyses among all randomised participants of the effects of allocation to fenofibrate versus placebo during the scheduled treatment period on the primary outcome in sub-categories of patient based on categories of data collected at baseline. Tests for heterogeneity of the proportional effect observed in subgroups will be used to determine whether the proportional effects in specific subcategories are clearly different from the overall effect.[2,3] (Note that for the subgroups that can be sensibly ordered [age, renal function and HbA1c] this test for heterogeneity is equivalent to a test for trend because each of the subgroups is to be split into only two categories [see Section 4.4.1.1].)

When many different subgroups are considered, chance alone may lead to there being no apparent effect in some subgroups considered in isolation, even when the effect of treatment really is about the same as is observed overall. In such circumstances, “lack of direct evidence of benefit” is not good “evidence of lack of benefit”, and clearly significant overall results would provide strong indirect evidence of benefit in subgroups where the results, considered in isolation, are not conventionally significant (or, even, perhaps, slightly adverse).[3-5] Hence, unless the proportional effect in some specific subcategory is clearly different from that observed overall, the effect in that subcategory is likely to be best estimated indirectly by applying the proportional effect observed among all patients in the trial to the absolute risk of the event observed among control patients in that category.[5]

7.4 Handling of missing and incomplete data

The study protocol and procedures have been designed to minimise loss to follow-up.[6] For any binary outcome event where the precise date of occurrence is unknown, the midpoint of the plausible range will be assumed (e.g. the middle of the month if only the month and year are reported). For subgroup analyses of the effects of fenofibrate on such events, patients with missing baseline values for that subgroup will be excluded from the subgroup-specific results but included in the overall total (with the result just among those with missing baseline data for each subgroup explicitly reported if the ‘missing’ category proves to be a non-trivial subset). Note that for analyses of the continuous measurements recorded at multiple follow-up time points, the MMRM models take account of missing data by assuming that such data can be predicted from the correlations seen between the non-missing values for other individuals, together with the other covariates in the model (i.e., the model assumes that data are ‘missing at random’).

7.5 Censoring schema for time-to-event endpoints

For all intention-to-treat analyses, participants who do not have the outcome under investigation will be censored at the end of the scheduled treatment period, which will be defined as the earliest of date of the final follow-up visit, death, or full consent withdrawal. For participants for whom information on these 3 criteria is missing, the censoring date will be set as the date when reliable information on both vital status and the occurrence/absence of the primary outcome was last reliably recorded (e.g. by completion of follow-up visit or access to relevant healthcare records).

8 Guidelines for early stopping or modification of the trial

Unblinded interim analyses will be reviewed regularly by the DMC, and will be conducted and presented by a statistician not involved in the day-to-day management of the trial. Upon review of such data, the DMC will advise the Steering Committee if, in their view, the randomised comparisons have provided both (a) proof beyond reasonable doubt that for all patients, or for some specific types, fenofibrate therapy is clearly indicated or contra-indicated; and (b) evidence that might reasonably be expected to influence materially the clinical management of many patients. The Steering Committee can then decide whether to end or modify the trial or to seek additional data, and this decision will be communicated in writing to the Chief Investigator after each Steering Committee meeting.

Appropriate criteria of proof beyond reasonable doubt cannot be specified precisely, but the DMC will work on the principle that a difference of at least 3 standard errors in an interim analysis of a major outcome event may be needed to justify halting, or modifying, a study before the planned completed recruitment. This criterion has the practical advantage that the exact number of interim analyses would be of little importance, and so no fixed schedule is proposed[3].

9 References

1. Cholesterol Treatment Trialists C, Baigent C, Blackwell L, et al. Efficacy and safety of more intensive lowering of LDL cholesterol: a meta-analysis of data from 170,000 participants in 26 randomised trials. *Lancet* 2010; **376**(9753): 1670-81.

2. Preiss D, Spata E, Holman RR, et al. Effect of Fenofibrate Therapy on Laser Treatment for Diabetic Retinopathy: A Meta-Analysis of Randomized Controlled Trials. *Diabetes Care* 2022; **45**(1): e1-e2.

3. Peto R, Pike MC, Armitage P, et al. Design and analysis of randomized clinical trials requiring prolonged observation of each patient. I. Introduction and design. *Br J Cancer* 1976; **34**(6): 585-612.

4. Peto R, Pike MC, Armitage P, et al. Design and analysis of randomized clinical trials requiring prolonged observation of each patient. II. analysis and examples. *Br J Cancer* 1977; **35**(1): 1-39.

5. Collins R, MacMahon S. Reliable assessment of the effects of treatment on mortality and major morbidity, I: clinical trials. *The Lancet* 2001; **357**(9253): 373-80.

6. Fleming TR. Addressing missing data in clinical trials. *Ann Intern Med* 2011; **154**(2): 113-7.

10 Appendix A: Details of randomisation algorithm

The minimised randomisation algorithm allocates participants to the placebo arm or the active arm. The participant is allocated to the treatment arm with the lower minimisation score, with a probability of 0.9 (i.e. this occurs for 90% of randomisations). For the other 10% of randomisations, or in the event of a tie (i.e. minimisation scores are equal), treatment is allocated randomly with a probability of 0.5.

The minimisation score is calculated based on the following categories:

- Sex: male; female
- Age at randomisation visit: <30; ≥30 <50; ≥50 <70; ≥70 years
- Type of Diabetes: Type 1, Type 2, Other
- eGFR from Randomisation assessment: <60; ≥60 mL/min/1.73m^2
- HbA1c from Randomisation assessment: <64; ≥64mmol/mol; unknown
- Statin therapy: Yes; No
- Most recent macula result: consider both eyes and use the worst eye's macula result as the category (in the list below, 'i' is the best result and 'iii' is the worst result)
    - i. No result available
    - ii. No maculopathy
    - iii. Observable diabetic maculopathy
- Most recent retina results: consider both eyes and use the worst eye's retina result as the category (in the list below, 'i' is the best result and 'iv' is the worst result)
    - i. Other retinopathy (this includes 'not adequately visualised' and 'other result provided')
    - ii. No retinopathy
    - iii. BDR – Mild
    - iv. BDR – Observable
